# Sympathetic tales: subdivisons of the autonomic nervous system and the impact of developmental studies

**DOI:** 10.1186/s13064-018-0117-6

**Published:** 2018-09-13

**Authors:** Uwe Ernsberger, Hermann Rohrer

**Affiliations:** Institute for Clinical Neuroanatomy, Goethe University, Theodor-Stern-Kai 7, 60590 Frankfurt/Main, Germany

**Keywords:** Sympathetic, Parasympathetic, Transcription factor, Preganglionic, Postganglionic, Autonomic nervous system, Sacral, Pelvic ganglion, Heart

## Abstract

Remarkable progress in a range of biomedical disciplines has promoted the understanding of the cellular components of the autonomic nervous system and their differentiation during development to a critical level. Characterization of the gene expression fingerprints of individual neurons and identification of the key regulators of autonomic neuron differentiation enables us to comprehend the development of different sets of autonomic neurons. Their individual functional properties emerge as a consequence of differential gene expression initiated by the action of specific developmental regulators. In this review, we delineate the anatomical and physiological observations that led to the subdivision into sympathetic and parasympathetic domains and analyze how the recent molecular insights melt into and challenge the classical description of the autonomic nervous system.

## Background

The “great sympathetic”... “was the principal means of bringing about the sympathies of the body”. With these words Langley [1, 2] summarized the ideas of Winslow [3] on connectivity and function of the intercostal nerve in his treatise on human anatomy. Within the general term “sympathetic” he included three nerves, the intercostal nerve or great sympathetic synonymous to the paravertebral sympathetic chain, the medium sympathetic synonymous to the par vagum or pair of vagus nerves and the small sympathetic synonymous to the portio dura of the seventh nerve. Thus, nearly two centuries before the large breakthroughs in physiological studies on nervous tissue and the synthesis of histological and cellular studies towards the neuron theory, the vagus and sympathetic nerves were linked by their naming and the assumption that these nerves are substantially involved in the matching of functional states among the different organs. This idea was taken up two centuries later by Cannon [4] in his monograph “The Wisdom of the Body” and the attempt to understand the interplay of nervous and hormonal control in particular mediated by the sympathetic nervous system and the adrenal gland in adapting the internal milieu to the changing external challenges the organism meets.

In this review we first describe the anatomical and physiological findings that led to the formulation of the classical model of the autonomic nervous system, subdivided into sympathetic and parasympathetic subsystems, acting partly in antagonistic manner. The heart as a prime target of autonomic innervation is discussed with respect to the historical unfolding of the physiological function of both autonomic nervous pathways regulating heart activity, their anatomical trajectories and the positions of the neuron cell bodies involved. We then consider the electrophysiological and neurochemical features of autonomic neurons, to illustrate neuron diversity even within each of the autonomic subsystems and to compare the cranial, thoracolumbar and sacral autonomic domains, their constituent cells and targets. This paves the way to delineate neuron development and factors regulating the acquisition of neuron subtype- specific features determining functional properties. We highlight transcription factor fingerprints of preganglionic and postganglionic neurons at different axial levels that suggest a sympathetic rather than parasympathetic developmental profile of the sacral spinal cord outflow, which stands in stark contrast to the classical model of autonomic neuron domains. Then we discuss the limitations of our understanding of the mechanisms responsible for the selective innervation of postganglionic neuron populations by the appropriate preganglionic neurons. Together with the more detailed characterization of a range of autonomic neuron populations so far underrepresented in the molecular and developmental analysis, a comprehensive understanding of the cellular composition and connectivity of the autonomic nervous system is expected to emerge.

## Main text

### Formulation of the classical model for the autonomic or involuntary nervous system at the turn to the 20th century

During the last two decades of the 19th century a series of keystone publications on structure and function of autonomic nerves were released from the Gaskell and Langley labs that provided the foundation for the thinking about the “autonomic” [1] or “involuntary” [5] nervous system dominating the 20th century.

Gaskell attempted to replace the nomenclature of the efferent nerves, which to him in part appeared entirely artificial or hypothetical, by fundamental divisions of the nervous system where physiological and structural properties can be grouped together. In a series of landmark papers on the nerves innervating the heart [6], the visceral and vascular systems [7] and the cranial nerves [8], Gaskell noticed the differences in the presence of small and non-myelinated (non-medullated) fibers in the nerves leaving the central nervous system from cranial to sacral levels. This histological approach to classify the efferent nervous system to the vascular and visceral muscles led to a subdivision into bulbar, thoracolumbar and sacral parts in addition to a small mesencephalic section [5]. A very similar conclusion was drawn by Langley [1] from a series of studies combining histological analysis, electrical stimulation, pharmacological intervention and nerve transection in the autonomic nervous system as exemplified by a series of treatises on the innervation of the pelvic and adjoining viscera [9–12]. The use of nicotine allowed the interruption of ganglionic transmission and the separation of preganglionic and postganglionic effects upon electrical stimulation of autonomic nerves. With the help of adrenaline and pilocarpine, as exemplified by the analysis of sweat gland regulation [13], muscarine and also choline, the effects of nerve stimulation could be compared to what then became known as noradrenergic and cholinergic neurotransmission. From a large set of data Langley divided the autonomic nervous system into a sympathetic, a parasympathetic and an enteric division [1]. The parasympathetic division is subdivided into a tectal, a bulbar and a sacral subdivision. While the effects of adrenaline closely resemble the majority but not all effects of sympathetic nerve stimulation, the effects of pilocarpine mimic the effects of parasympathetic nerve stimulation.

For Langley [1, 14] it appeared convenient to cover the tectal and the bulbo – sacral nerves under the name parasympathetic since they differ in their pharmacological response from the sympathetic nerves whose action in general can be mimicked by application of adrenaline. He was eager to point out, however, that the sweat gland regulation by sympathetic nerves poses an exception as this target tissue responds to pilocarpine similar to parasympathetic target tissues. In addition, Langley emphasized that sympathetic nerves supply all parts of the body while the parasympathetic nerves supply only special parts.

An important issue in Langley’s synthesis [1] is the discussion of the source of the preganglionic axons to the sympathetic and parasympathetic system. He emphasized the critical contribution by Gaskell [5, 7] on the distribution of myelinated fibers in the communicating rami, pointing out the gaps in their presence between thoracolumbar levels and cranial as well as sacral levels. This is nicely illustrated by Gaskell [7] in his classical publication on the white and gray rami communicantes and the visceral nerves in particular in dogs as well as by Pick and Sheehan [15] presenting a drawing of the macroscopic situation in man from cervical to sacral levels and cross-sections on individual rami communicantes.

### Integration of knowledge into the classical model of the autonomic nervous system during the 20th century

The classical model of the sympathetic and parasympathetic nervous system provided an amazingly constructive framework for results coming in from the biomedical disciplines at increasing speed. The division into two subsystems acting at least in part in an antagonistic manner based on two neurotransmitter systems provided a very attractive framework for considering system biological problems and to confront a vast range of therapeutic challenges. The opposite action of sympathetic and parasympathetic stimulation on the ciliary muscle, the heart and the reproductive organs were but three examples where the attraction of this approach became apparent. Histological, electrophysiological, pharmacological and neurochemical approaches became the main motors to complete an anatomical and physiological description of cellular structure and function of the autonomic nervous system and its target structures [16, 17] as well as an increasing understanding of the dysfunctions.

#### Reciprocal regulation of heart activity by vagus and sympathetic nerves

The interrogation of the neural control of the heart at the turn of the 19th century resolved the problem of whether the heart was able to move independently of the presence of the nervous system and the question for the contribution of the nervous system to the modulation of heart activity [18]. Work on the effects of nerve transection on heart activity that had been focusing on the vagus and intercostal nerves was complemented by electrical stimulation experiments inspired by Galvani’s and Volta’s observations [19]. During this time, the consensus developed that the heart is operating autonomously yet the operation can be modified essentially by the vagus and intercostal nerve.

The first convincing report on antagonistic regulation of the heart activity by the vagus and the sympathetic nerve is attributed to the brothers Ernst Heinrich and Eduard Weber using the electromagnetic rotation apparatus for experiments performed in frogs and confirmed in birds and mammals. Ernst Heinrich Weber reported in 1845 (as cited by von Bezold, 1863) that galvanic excitation of the vagus nerve weakens the heart and slows down or interrupts the heartbeat, while excitation of the sympathetic restores, enhances and enforces the movement of the heart.

At the time Langley and Gaskell released their key papers, Bayliss and Starling [20] published an influential set of experiments on the autonomic regulation of mammalian heart function performed in dogs. Stimulation by induced currents of the parasympathetic vagus nerve and the sympathetic cardiac branches originating from the inferior cervical ganglion demonstrated cardio – inhibitor and cardio – accelerator effects, respectively, on heart rate as well as output. Likewise in dogs, analysis of stimulation and resection of vagal nerves and parts of the sympathetic strand were performed and analyzed in the exercising animal [21, 22] to demonstrate the opposite effects of parasympathetic and sympathetic stimulation on heart rate and blood pressure.

A quantitative model for the regulation of heart rate by the parasympathetic – sympathetic antagonistic action was developed [23], yet the complexity as well as incompleteness of this quantitative understanding was appreciated [24]. The characterization of the precise trajectories of the autonomic innervation to the heart culminated in the compilations of the experimental results on the neuronal elements involved in the neuronal control of the heart [25, 26] founding the discipline of “Neurocardiology” [27]. The reciprocal and nonreciprocal components of the interaction of parasympathetic and sympathetic effects on heart beat under “normal” and “non-normal” conditions were demonstrated [28]. For a range of reasons heart rate variability and the effects of the autonomic nervous system and other players thereon remained of particular interest [29]. To understand the neural networks subserving the autonomic regulatory circuits, the trajectories of the parasympathetic and sympathetic fibers and their reflex regulation by relevant sensory stimulation were analyzed.

The anatomical course and physiological impact of the cardio – inhibitor and cardio – accelerator fibers were studied in different mammalian species. They are exemplified by studies in dogs where electrical stimulation and surgical interruption of different cardiac nerve branches and the paravertebral sympathetic trunk were combined to determine the course of the preganglionic vagal and postganglionic sympathetic neurons [30–32]. Postganglionic parasympathetic neurons in the heart were described later [33–35] and the understanding of their organizational structure was refined to the current level [36]. Crucial progress in the understanding of the functional organization of this system emerged in the 1970s. The autonomic control of the heart by sympathetic and parasympathetic nerves was analyzed at the level of reflex regulation of nerve activity by chemoreceptor and baroreceptor stimulation [37–39] including the characterization of the activity patterns of identified preganglionic and postganglionic sympathetic neurons [40, 41]. The comparison of reflex regulation of sympathetic and parasympathetic nerve activity in relation to the heart rate was investigated under atrial distention in the dog [42–44] and a similar analysis performed with chemoreceptor activation [45, 46].

The balanced interaction of the two tracks of the autonomic nervous system during regulation of heart function as reflex action under different stimulus settings and stressor regimes and its relation to heart dysfunction and autonomic conflict remain the focus of continuing interest [47, 48]. Understanding the sympathetic parasympathetic antagonism and the applicability of the Rosenblueth – Simeone model became addressed during human exercise [49, 50]. Under baroreflex stimulation and pharmacological blockade the alterations in sympathetic and parasympathetic activities under exercise were estimated [51]. Of particular interest, the imbalance of the autonomic nervous system in the pathophysiology of heart failure and infarction remains a key question [52–54]. Here, the functional reorganization of the parasympathetic and sympathetic innervation to the heart has attracted considerable interest [55].

#### Tracing of the sympathetic and parasympathetic connections to the heart

Even though the anatomical connection of the vagus nerve, the sympathetic trunk and the heart was already recognized in the 18th century [2], anatomical studies to compare heart innervation among human subjects and monkeys have remained of interest until the 21st century, yet at submicroscopic scales [56]. A critical contribution to the understanding of autonomic target innervation at the cellular level came from retrograde labeling studies in particular with the help of horseradish peroxidase (HRP) application to the target tissues or sectioned nerve endings. A more recent study [57] analyzed neurons labelled by HRP application to the heart of monkeys. The detection of labeled neurons in the nucleus ambiguus of the brainstem as well as the superior and medium cervical and stellate ganglia of the longitudinal sympathetic strands demonstrates that this labeling discloses preganglionic vagal and postganglionic sympathetic neurons.

Labeling of the entire vagus nerve or the target structures heart, lungs and stomach was performed in the cat by Kalia [58] and disclosed the positions of the somata of the preganglionic neurons in the nucleus ambiguus, the dorsal motor nucleus of the vagus and the nucleus retroambiguus. Application of HRP into the sinoatrial or ventricular myocardium of the rat labeled cells primarily in and around the nucleus ambiguus [59]. Upon injection of HRP into physiologically identified cardio – inhibitory filaments of individual cardiac nerves, which elicited bradycardia and negative inotropism after stimulation, this location was confirmed and directly linked to the physiological action of the nerves analyzed [60].

Upon HRP application to the heart and aortic arch of the dog, the greatest number of labeled postganglionic cell bodies is detected in the medium cervical ganglia, in addition in the cranial poles of the stellate ganglia and occasionally in the superior cervical ganglia [61]. Upon HRP injection at different sites in the heart of the cat, sympathetic neurons were predominantly localized in the right stellate ganglion, fewer in the superior and medium cervical ganglia and fewest in thoracic ganglia [62]. The comparison to the observations in monkeys, where most of the cells are found in the superior cervical and less in the stellate ganglion [57] indicates that the major site of postganglionic sympathetic neuron somata to the heart may differ between mammalian species.

#### Characterization of the sympathetic neurons at different levels along the rostrocaudal axis

The connectivity between preganglionic and postganglionic neurons was analyzed by electrophysiology in the superior cervical ganglia of the guinea pig [63]. This provided detailed understanding of the segmental distribution along the cervical and thoracic spinal cord of preganglionic neurons synapsing onto individual postganglionic neurons, the number of innervating preganglionic neurons terminating onto individual postganglionic neurons and the strength of the connections. However, the precise target of the studied postganglionic neuron remained unknown [64]. A study of the innervation of postganglionic neurons in the celiac ganglion also by intracellular recording provided a classification of neurons with different responses to preganglionic stimulation [65]. Again, the precise targets of the analyzed cells could not be revealed from the isolated ganglion preparation.

Characterization of the electrophysiological properties of preganglionic sympathetic neurons and their reflex regulation by sensory stimuli demonstrated a diversity of neuron populations that may subserve different functions [66–68]. This was also reflected in the reflex regulation of the activity of postganglionic sympathetic neurons by different sensory stimuli (Table 1). Distinct alterations in neuronal activity patterns were observed upon extracellular recording in nerve branches preferentially innervating distinct sympathetic targets such as skin or muscle vasculature or sweat glands [69–71]. These and subsequent studies indicated that different sympathetic pathways are available to different target organs and tissues [72] to mediate homeostatic orchestration of target functions [73].

**Table 1:** Selected subpopulations of postganglionic sympathetic neurons

A) electrophysiologically defined subpopulations							
Neuron class	MVC	CVC	SM		PM		
Transmitter	NE	NE	ACH		NE		
Peptide cat	NPY	NPY	VIP				
	GAL	GAL	CGRP		GAL		
Peptide guinea pig	NPY	NPY	VIP				
		DYN	CGRP		DYN		
major stimulus							
human	baro-inhibition	cooling general	warming general				
cat	baro-inhibition	temperature	vibration		hypothalamic stimulation	(selected from [17]	
B) subpopulations defined by RNA sequencing							
Neuron class	NA 1	NA 2	NA 3	NA 4	NA 5	ACH 1	ACH 2
Average transcript number per cell							
TH	69	105	93	85	100	25	1
DBH	48	83	71	79	67	37	21
DDC	43	105	91	107	88	28	12
VMAT 2	29	63	58	38	34	4	2
CHAT	zero	zero	zero	zero	zero	2	1
VACHT	zero	zero	zero	zero	zero	7	9
NPY	117	678	478	63	22	74	11
SOM	zero	1	zero	zero	zero	3	53
VIP	1	1	1	zero	zero	367	200
CGRP (CALCA/B)	zero	zero	zero	zero	zero	4/3	6/5
defined target		erector			erector		
		muscle			muscle		
compiled from [80], supplementary figure nn.4376 – S4							

The table displays a selected set of sympathetic neurons derived from electrophysiological analysis (A) or from RNA sequencing profiles (B)

Electrophysiological analysis (A) defined a large number of sympathetic neuron classes named according to the target tissue supplied by the nerves from which recordings are made: *MVC* Muscle vasoconstrictor, *SVC* Skin vasoconstrictor, *SM* Sudomotor and *PM* Pilomotor among other populations not listed here. Classical neurotransmitters *NE* Norepinephrine and *ACH* Acetylcholine as well as neuropeptides detected in cat and guinea pig are provided for the individual neuron classes. In addition, the major stimuli detected by microneurography in humans and extracellular recording from prepared nerve filaments in cats are indicated to demonstrate the different reflex circuits and functional integration of the neuron classes

RNA sequencing profiles analyzed by unsupervised clustering algorithms (B) from material derived from stellate and thoracic mouse sympathetic ganglia disclosed a number of noradrenergic (NA 1 to 5) and cholinergic (ACH 1, 2) neuron populations distinguished by the preferential expression of certain genes. The numbers shown for the different genes give the average number of transcripts for the respective gene in a cell of a given population. Interestingly transcripts for noradrenergic markers *TH* Tyrosine hydroxylase, *DBH* Dopamine beta hydroxylase, *DDC* DOPA decarboxylase and the *VMAT 2* Vesicular monoamine transporter 2 are not absent from the cholinergic neuron populations. On the other hand, cholinergic markers *CHAT* choline acetyltransferase and the *VACHT* Vesicular acetylcholine transporter are not detectable in the noradrenergic neuron populations. The *NPY* Neuropeptide is not absent from cholinergic neurons while *SOM* Somatostatin and *VIP* Vasoactive intestinal polypeptide are largely restricted to one or both cholinergic neuron populations. The targets given for the NA 2 and NA 5 are derived from developmental analysis and genetic labeling of specifically expressed genes. The high level SOM expression in ACH2 is characteristic for sudomotor neurons

Characterization of the electrophysiological properties in combination with morphometric analysis and histochemical classification [74] complemented the understanding of the nature of sympathetic postganglionic neurons. Comparison of animal studies with microneurography in humans confirmed that sympathetic postganglionic neuron populations characterized in mammalian model organisms also can be detected in humans [75].

**Fig. 1:**
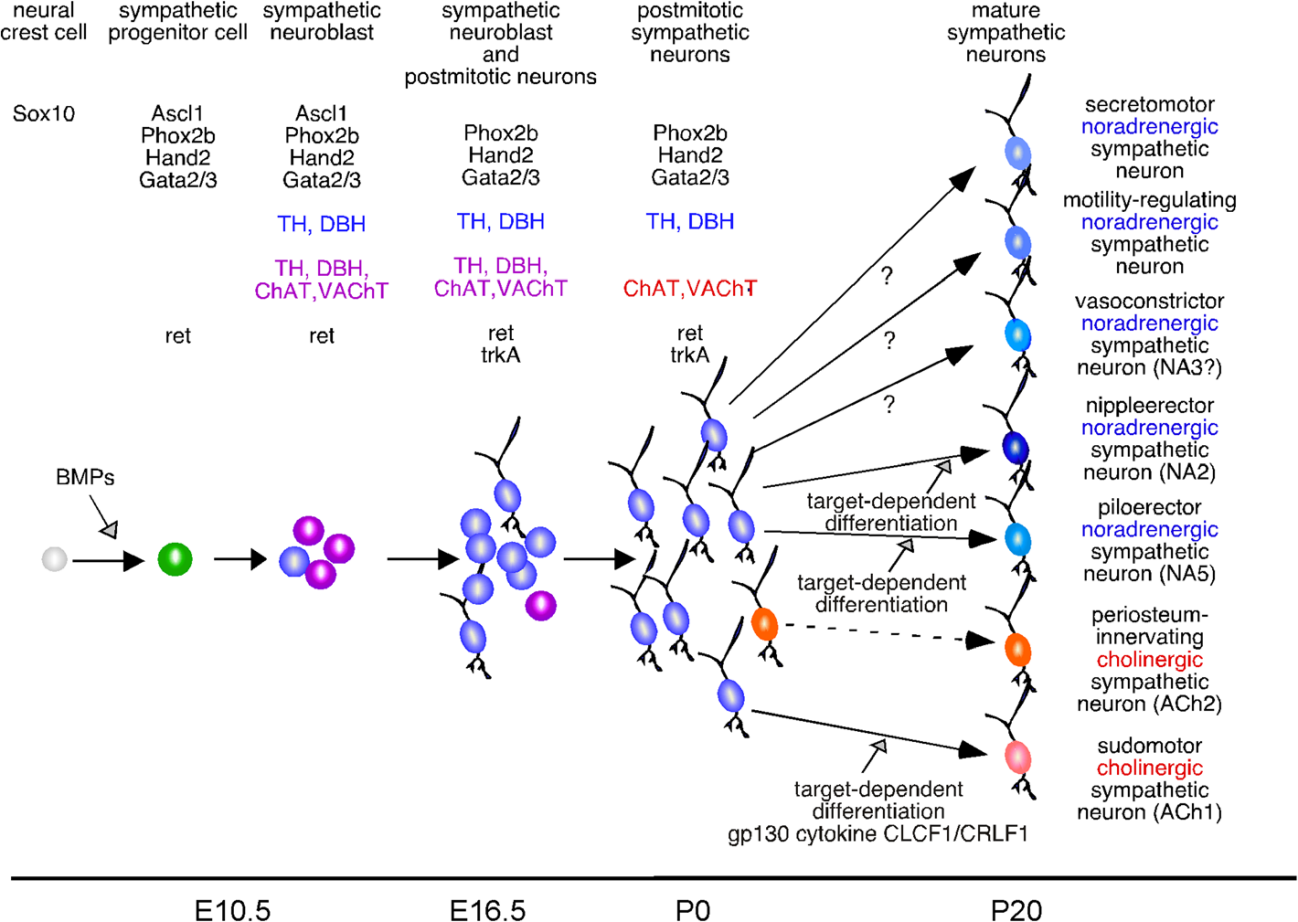
Schematic illustration of the sympathetic neuron subtype differentiation in the mouse. BMP-signaling at the dorsal aorta elicits the expression of a group of transcription factors, including Phox2b, Hand2 and Gata3 [156–158, 221] that induce noradrenergic (Th, Dbh) and cholinergic genes (ChAT, VAChT), resulting in a high proportion of cells with a mixed noradrenergic/cholinergic phenotype at E10.5-E11.5 [143, 151]. At birth, the vast majority of postmitotic sympathetic neurons display noradrenergic properties; cholinergic characteristics are observed only in about 5% of sympathetic neurons [80, 151, 222]. Single-cell RNAseq of mature sympathetic neurons from P30 sympathetic ganglia allowed to define 2 subtypes of cholinergic sympathetic neurons (ACh1 and ACh2) (labeled by red cell bodies) and 5 subtypes of noradrenergic sympathetic neurons (NA1–5) (noradrenergic sympathetic neuron subtypes are labeled by different shades of blue) [80]. ACh1 and ACh2 correspond to previously identified sudomotor and periosteum-innervating neurons [85, 153]. NA2 and NA5 have been identified as nippleerector and piloerector sympathetic neurons. Sudomotor, NA2 and NA5 subtypes differentiate during postnatal development from noradrenergic neurons under the influence of target-derived differentiation signals [80, 87]. Vasoconstrictor, secretomotor, motility-regulating sympathetic neurons as well as other subtypes identified by physiological approaches are not yet characterized with respect to their gene expression signature and whether their differentiation is also controlled by target-derived signals

In addition to the location of the cell bodies of the autonomic neurons, their histological characterization provided increasing insight into their nature. In particular the neurons of the sympathetic ganglia became the subject of histological and molecular analysis that provided insight into the neurotransmitter phenotype [76–78], their neuropeptide complement [79] and, in very recent times, their entire transcriptome [80]. The introduction of immunohistochemistry and later in situ hybridization beautifully demonstrated that the vast majority of sympathetic neurons, which physiologically and pharmacologically were characterized noradrenergic, were distinguished by catecholamine histofluorescence [81], expression of the enzymes required for noradrenaline biosynthesis [82] and coexpression of all the genes coding for the required enzymes in addition to transporter proteins involved in catecholamine uptake and storage [83]. Yet cholinergic neurons were also found [84, 85] as is expected from physiological studies. In the stellate ganglia of rodents they constitute a small (about 5%) but significant population, which is established during postnatal development under the influence of the target tissues, in particular sweat glands [80, 86, 87]. Quantitative gene expression analysis in individual cells of cervical and thoracic ganglia allows the identification of subpopulations of sympathetic neurons targeted to different tissues and the characterization of the gene products determining the physiological properties of these neurons [80] (Fig. 1).

Such a detailed knowledge is not yet available for preganglionic sympathetic or pre– and postganglionic parasympathetic neurons. Characterization of the postganglionic vagal neurons innervating the heart is still incomplete. Histochemical characterization of the heart ganglia demonstrated the presence of a cholinergic neuron population, considered to represent the postganglionic parasympathetic neuron population, and a population of small intensely fluorescent cells whose function is not fully characterized [88]. The comparison of their gene expression profile to that of sympathetic neurons in the cervical and stellate ganglia will be of prime interest.

Of similar interest will be the characterization of the preganglionic neurons in the sympathetic and parasympathetic systems. A very important finding was the discovery of the Phox2 transcription factors expressed in preganglionic motoneurons of the brainstem [89]. In the respective mutant mice cranial preganglionic neurons in the dorsal motor nucleus of the vagus and nucleus ambiguus are missing already in embryonic development. In contrast, somatic and visceral spinal cord motoneurons are generated from Olig2-expressing progenitors and are characterized by Hb9 and Islet1 transcription factors [90, 91].

#### Autonomic innervation of the abdominal viscera

Of note, application of HRP to the vagus nerve not only labels preganglionic parasympathetic neurons in the brainstem but also postganglionic sympathetic neurons in the cervical sympathetic ganglia. Even HRP application to the cervical vagus reveals labeling in the sympathetic trunk [92]. Moreover, innervation to the abdominal viscera via the vagus nerve can be traced to the cervical and thoracic ganglia [93].

After HRP application to the duodenum and jejunum in the cat and the guinea pig, sympathetic neurons are not only labeled in the celiac ganglion but also in the cervical and stellate ganglia of the sympathetic trunk [93]. Since crushing of the vagus nerve deletes the labeling in the cervical and stellate ganglia, their postganglionic neurons to these targets project in the vagus nerve. Further distal parts of the gut as well as pelvic viscera are innervated by different domains of the autonomic nervous system involving lumbar sympathetic postganglionic neurons and sacral, initially called parasympathetic, preganglionic neurons.

Thus, the abdominal parts of the digestive tract are innervated by several domains of the sympathetic and of the parasympathetic nervous system. The cervical and thoracic domains of the sympathetic system target the duodenum and jejunum via postganglionic neurons from cervical and thoracic ganglia running in the vagus nerve. In addition, neurons from the celiac ganglia and the splanchnic nerves are involved. Lumbar domains of the sympathetic nervous system target the colon via postganglionic neurons in colonic nerves from the mesenteric ganglia [94–96]. The lumbar sympathetic domains also target the remaining pelvic viscera [97]. This abdominal region is also innervated by autonomic neurons originating in the sacral spinal cord [98, 99]. Preganglionic neurons can be labeled by HRP application to the pelvic and pudental nerves [100–104].

Postganglionic sympathetic neurons innervating abdominal viscera are located in the paravertebral chain of ganglia, in prevertebral ganglia, in isolated clusters of neurons found in the aortic plexus and plexuses accompanying arterial vessels as well as the superior hypogastric plexus, and in the pelvic ganglion or pelvic plexus as it is called in species where the condensation into a well demarcated ganglion is not so prominent.

The prevertebral ganglia – the celiac, superior and inferior mesenteric ganglia – were subject to morphological, neurochemical and electrophysiological characterization as described for the cervical and thoracic sympathetic ganglia [65, 74, 105–107]. A genome wide transcriptome analysis is not yet available, however.

The pelvic ganglion or plexus is unique due to its dual composition [108] reflected in the presence of large numbers of cholinergic in addition to noradrenergic neurons [109–111] and their largely but not entirely selective innervation by preganglionic neurons from the sacral and lumbar spinal cord, respectively.

#### Summarizing remarks

Fostered by the remarkable progress in electrophysiological instrumentation, histochemical techniques and pharmacological approaches, research on the autonomic nervous system in particular during the second half of the 20th century established detailed knowledge of the cellularity and connectivity of the sympathetic and parasympathetic system. The technique of retrograde neuronal tracing by HRP application to sectioned nerves or into target tissues provided a breakthrough in the localization of preganglionic and postganglionic autonomic neurons. Extracellular recordings allowed the characterization of neuronal behavior under control and experimental conditions thus demonstrating the presence of different populations of preganglionic and postganglionic neurons providing pathways to distinct target tissues as exemplified by the sympathetic supply of different vascular beds and other targets. Intracellular recording techniques provided access to the electrical properties of neurons and again demonstrated the presence of different populations of postganglionic neurons in the accessible sympathetic ganglia. In addition they allowed the study of synaptic input from preganglionic neurons to address questions concerning the synaptic integration of autonomic neuronal activity. With these and other techniques a detailed picture of the cellular structure of the autonomic nervous system was developed for adult mammals and assembled into a model of functional organization of two partly antagonistic systems mediating a homeostatic control of key organs.

### Challenges to the classical model of the autonomic nervous system from development studies at the beginning of the 21st century

During the first half of the 20th century the basic science approach towards the autonomic nervous system was largely restricted to mature mammalian organisms such as cats and dogs. This changed dramatically since the 1950‘s. The discovery of nerve growth factor (NGF) has put the development of postganglionic sympathetic as well as primary sensory neurons at center stage of Developmental Neuroscience. In the 1970s, immunohistochemical techniques provided specific access to the rate limiting enzyme of catecholamine biosynthesis. Developmental analysis at that time focused on postganglionic sympathetic neurons, expression of the noradrenergic marker enzyme tyrosine hydroxylase and the role of neurotrophic factors in their development. During the 1990s, this focus was considerably strengthened and widened by in situ hybridization for mRNA detection in developing tissues and the inclusion of different markers of neuron populations such as several enzymes of the noradrenaline biosynthesis cascade and transporter proteins. Growth factor receptor protein subunits and transcription factors responsible for cell type specific gene expression and its regulation during development could be analyzed. At the turn of the 21st century, this approach in combination with viral overexpression studies in chick embryos and mutational inactivation studies in mice provided critical insight into the differentiation of the noradrenergic transmitter phenotype in sympathetic neurons and the growth factors and transcription factors involved. In recent years this analysis has been extended to compare sympathetic neuron development at thoracolumbar levels to parasympathetic neuron development at cranial levels. In addition, autonomic neuron development was studied at sacral levels. This resulted in the critical finding that both preganglionic as well as postganglionic neurons at sacral levels are related in the regulation of their differentiation with thoracolumbar sympathetic but not cranial parasympathetic neurons.

#### Nerve growth factor (NGF) and the onset of developmental analysis into the sympathetic nervous system

The discovery and characterization of NGF and its action on peripheral neurons initiated the molecular interrogation into the sympathetic nervous system [112–114]. Different from sympathetic neurons parasympathetic neurons of the ciliary ganglion appear not to depend on NGF [115]. The interference with nerve growth factor action by application of antisera in mammalian embryos and adult animals proved the requirement for this growth factor for sympathetic neuron development. This was beautifully complemented by the inactivation of the NGF gene in mice resulting in the loss of sympathetic ganglia [116] as observed with the inactivation of the gene coding for the high affinity NGF receptor trkA [117]. The loss of sympathetic neurons in trkA mutant mice becomes apparent after the second embryonic week in mice shortly after onset of trkA expression [118].

Uncoupling of the survival function of NGF by deletion of the proapoptotic Bax2 gene allowed the analysis of additional functions in vivo [119]. The findings in mice demonstrated that the innervation of different target tissues is affected at very different levels, some targets receiving no supply by noradrenergic sympathetic neurons and some receiving almost the normal complement of tyrosine hydroxylase (Th) –positive fibers. In addition to the importance of NGF for innervation of sympathetic target tissues, this growth factor is critical for development of the dendritic arbor of the neurons [120]. In addition, the development of the synaptic connections between preganglionic and postganglionic sympathetic neurons depends on NGF [121] where activated trkA receptors are required in the dendritic compartment of the postganglionic neurons to maintain the synaptic connections with the preganglionic input [122] (see also below).

#### Histochemical and biochemical methods to assess noradrenergic neurons

The introduction of a range of histochemical and biochemical techniques in the second half of the 20th century boosted developmental analysis of autonomic neurons with particular emphasis on sympathetic neurons. Accessibility of catecholamines and the rate limiting enzyme in their synthesis, tyrosine hydroxylase (Th), by formaline– induced histofluorescence, enzyme activity measurements and immunohistochemistry established Th as phenotypic marker for catecholaminergic neurons, their biochemical activity state, protein expression and gene transcription. The histofluorescence technique [123] was applied to sympathetic neuron development in chick to describe the formation of the sympathetic strands [81, 124] from migrating neural crest cells [125]. This was extended by the analysis of Th activity and immunoreactivity in sympathetic neurons of embryonic chick [126], rat [127–129] and mouse [130] embryos.

A remarkable advantage of the histofluorescence technique was the possibility for combination with nucleolus staining in “quail-chick” chimeras resulting in the breakthrough in the analysis of the neural crest contribution to the postganglionic sympathetic and parasympathetic system [131]. Transplantation of neural tube from somite stage quail to chick embryos and analysis of the distribution of donor cells in peripheral ganglia of those animals demonstrated that sympathetic neurons are recruited from somitic levels 8 to 28 while levels 1 to 7 contribute neurons to parasympathetic ganglia [132]. Detailed analysis with transgenically labeled chick demonstrates that somitic levels 3 to 7 in addition to their parasympathetic contribution provide neurons to the superior cervical ganglion [133].

Another critical finding was the observation that sympathetic neuroblasts are still able to divide after noradrenergic and neuronal differentiation. Combination of catecholamine detection by histofluorescence techniques and demonstration of cell division by incorporation of radiolabeled thymidine into newly synthesized DNA demonstrated that sympathetic neuroblasts in chick embryos are still dividing when they have already acquired neuronal and noradrenergic properties [134, 135]. This marks a difference to parasympathetic neurogenesis as observed in ciliary ganglia [136]. A detailed kinetic analysis of cell division and differentiation in postganglionic sympathetic neurons of mice and chick has been obtained by the comparison of incorporation of two labeled nucleotides, BrdU and EdU, into the newly synthesized DNA [137, 138]. The studies show a temporary deceleration or complete interruption of cell cycling only to resume proliferation after differentiation to the noradrenergic phenotype.

#### Induction of the noradrenergic transmitter phenotype in sympathetic neurons

With in situ hybridization a highly specific as well as selective method was introduced to allow detection of gene expression by mRNA labeling in tissues and even single cells. Comparison of gene expression onset for noradrenergic markers and transcription factors demonstrates an early onset of Th and dopamine beta – hydroxylase (Dbh) transcript detectability following the paired homeodomain protein transcription factors Phox2a and Phox2b in mice and in chick [82, 139, 140]. Mutational analysis in mice demonstrates that Phox2b is indispensable for postganglionic sympathetic neuron development, yet also is required for the generation of postganglionic parasympathetic neurons [140]. As such it marks a key transcriptional regulator for the generation of all postganglionic neurons in the sympathetic and parasympathetic branch of the autonomic nervous system. In parallel the surprising observation was made that postganglionic sympathetic neurons early during development did not only express noradrenergic markers such as Th and Dbh but also the cholinergic locus genes choline acetyltransferase (ChAT) and the vesicular acetylcholine transporter (VAChT) [141–143] (Fig. 1). In chick as well as mouse embryos Phox2b is required for induction of expression of both gene sets.

**Fig. 2:**
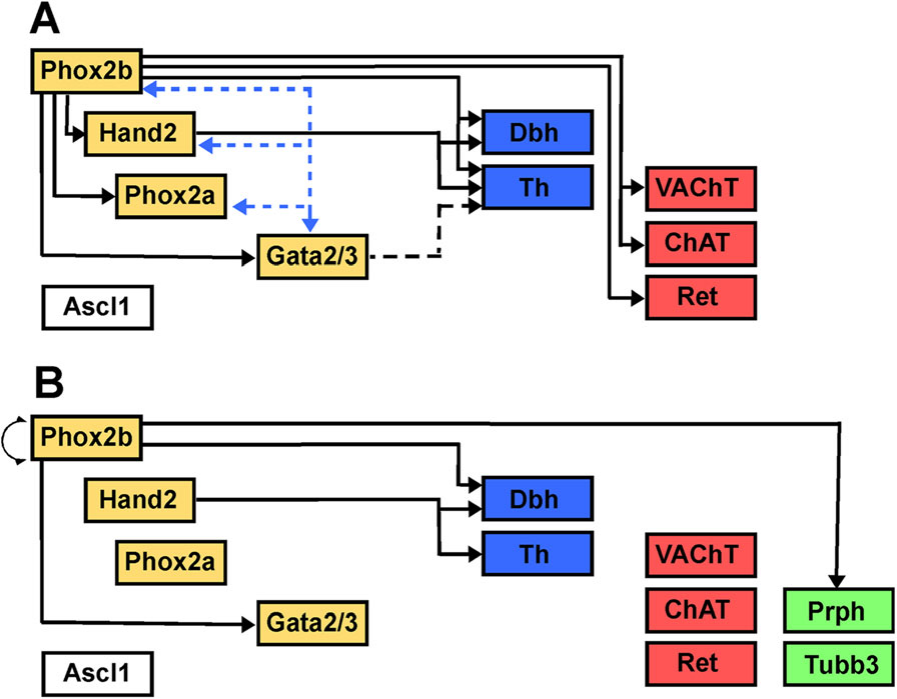
Transcriptional control of sympathetic neuron development. **a** Target genes regulated by Phox2b in sympathetic progenitors are detected in the Phox2b-knockout mouse [140]. Solid black arrows indicate complete absence of the indicated target genes in mutant embryos. In addition to the noradrenergic marker genes Th and Dbh (noradrenergic genes labeled by blue boxes), the cholinergic markers ChAT and VAChT (cholinergic genes labeled by red boxes) are not expressed in Phox2b mouse mutants [142]. Phox2b does not control initial expression of Ascl1 (white box). Expression of the transcription factor Hand2, which is required for Th and Dbh expression [223] depends on Phox2b [224]. Expression of Gata3, which increases Th transcript levels also depends on Phox2b [173]. Embryonic overexpression demonstrates that each of the transcription factors Phox2b, Phox2a, Hand2 and Gata3 is able to induce the expression of any of the other factors in progenitor cells [173, 225, 226] (blue stippled arrows). **b** In differentiated neurons different target genes are regulated by Phox2b as detected in conditional mutant mice deleting Phox2b after initial differentiation [152]. In differentiated sympathetic neurons Phox2b enhances its own expression but is dispensable for Phox2a and Hand2 yet remains required for Gata3 expression. Markers for the cholinergic phenotype as VAChT, Vip and Ret (cholinergic genes labeled by red boxes) appear independent of Phox2b, as well as the generic neuronal marker Tubb3 (generic neuronal genes are indicated by green boxes). On the other hand, peripherin (Prph) and Dbh depend on Phox2b. Hand2 remains required for Th and Dbh expression (noradrenergic genes labeled by blue boxes) in differentiated embryonic sympathetic neurons [149]. Notably, Hand2 elimination in adult sympathetic neurons reveals still another set of target-genes involved in synapse function [147]

Gene overexpression studies in chick embryos as well as mutational inactivation in mouse embryos demonstrates that a set of transcription factors including Phox2a and 2b, Gata2 and 3, Hand1, 2 and 3 and Ascl1 interact as a network to accomplish sympathetic neuronal differentiation [144, 145] (Fig. 2a). An important difference in the transcription factor network between sympathetic and parasympathetic postganglionic neurons observed here concerns Hand2 [146, 147], the homeo box transcription factor HoxB8 [148] and the transcription factor Hmx1 [143]. Hand2 in mouse, chick and zebrafish sympathetic neurons is found to promote noradrenergic differentiation [149, 150]. Hmx1 mutation in mouse embryos compromises the expression of trkA and the maintenance of Th transcripts. In addition, it prevents the downregulation of the ret receptor subunit involved in the expression of cholinergic properties [143].

#### Generating diversity among postganglionic sympathetic neurons

While noradrenergic and cholinergic properties are co-expressed during early differentiation in postganglionic sympathetic neurons in both avian and rodent embryos, cholinergic properties become downregulated rapidly in the majority of the cells while noradrenergic properties remain prominently expressed [141, 151]. Interestingly, cholinergic gene expression is not controlled by Phox2b in differentiated sympathetic neurons [152] (Fig. 2b). Moreover, in mice many cells of the small population of cholinergic neurons retain expression of genes such as Th and Dbh [83]. Ret, the signal transducing subunit of the GDNF family ligand receptors, is required for the embryonic development of cholinergic sympathetic neurons [151] and is negatively regulated by Hmx1 [143]. The majority of cholinergic sympathetic neurons in mice, however, acquire their identity by a switch from a noradrenergic to a cholinergic phenotype after birth of the animals mediated by induction of cholinergic properties in previously noradrenergic neurons by the interaction of the outgrowing nerve fibers with selected target tissues such as sweat glands and periosteum [85, 86, 153] and neurokine signaling via the gp130 receptor is required [87, 222] (Fig. 1). Whether the cholinergic phenotype in postganglionic parasympathetic neurons is brought about in a manner similar to sympathetic neurons and how the transcriptome looks in the parasympathetic neurons is currently unclear, besides the absence of Gata3 and Hand1 and the specific expression of Hmx2 and Hmx3 [154]. It should be mentioned, however, that embryonic postganglionic parasympathetic neurons also transiently express noradrenergic characteristics [146, 147, 155].

RNA sequencing from individual sympathetic neurons in stellate and thoracic ganglia in mice has now allowed to characterize multiple populations of postganglionic sympathetic neurons by their gene expression profile [80]. In this manner several classes of noradrenergic neurons could be distinguished apart from a small cholinergic neuron population. Transgenic labeling of individual populations allowed the identification of piloerector and nippleerector neurons and demonstrated their full differentiation at postnatal stages due to interaction with the target tissue (Fig. 1). Postganglionic neurons innervating the heart are still not identified preventing genetic targeting and manipulation of this population. The approach chosen by Furlan [80] can also be expected to specify the transcriptome of physiologically defined postganglionic sympathetic neuron populations as they are described by Jänig [17] to unify molecular and electrophysiological classifications.

#### Development of the synaptic machinery in postganglionic autonomic neurons

The postganglionic autonomic neuron is the final element in the autonomic neural circuit and responsible for integration of the central neural input and information propagation to the target tissue. The postsynaptic apparatus receiving the pre-synaptic input in the autonomic ganglion and the presynaptic apparatus conveying the activity pattern at the target synapses are complex machineries crucial for the selective handling and propagation of the information content. Studies in chick ciliary and sympathetic ganglia have shed some light on their development and function.

It is noteworthy that the initial development of neurons in chick ciliary and sympathetic ganglia shares many features. Bone morphogenetic proteins (BMPs) are required for the initiation of differentiation in both systems [146, 156–158]. Growth factor action in both ganglia initiates a highly similar sequence of transcription factor expression with the notable exception of Hand2 expression found in chick sympathetic but not ciliary ganglia, followed by the sequential initiation of noradrenergic and cholinergic gene expression [139, 141, 146]. Noradrenergic and cholinergic genes are also transiently co-expressed in mouse embryonic sympathetic and parasympathetic neurons [143, 147, 152, 155].

With short delay after initiation of the noradrenergic marker gene induction and several days before target tissue innervation expression of synaptic protein genes commences in chick sympathetic ganglia. This is shown for synaptotagmin I, a critical calcium sensor of the transmitter vesicle membrane, and neurexin 1, a crucial organizer of protein complexes within the pre-synaptic membrane and binding partner to post–synaptic neuroligins [159]. During the time of target innervation, neurexin isoform expression is altered [160]. Also in the chick ciliary ganglion several neurexin isoforms are expressed and are able to induce acetylcholine receptor clustering [161]. Synaptotagmin and synaptophysin are detected before target innervation in ciliary ganglia and isoform expression changes during target innervation [162]. In addition, regulation by synaptic inputs from preganglionic fibers is shown to affect synaptic protein gene expression [163]. Interestingly, synaptotagmin II is the prevailing isoform in pre-hatching chick ciliary ganglia while it is absent in sympathetic ganglia where synaptotagmin I is strongly expressed. The data show that synaptic gene expression is induced early during neuronal differentiation and subsequently regulated in a complex manner by target tissue and preganglionic innervation.

The development of synapses and transmission in chick ciliary ganglia has been investigated by electrophysiological and ultrastructural analysis [164]. Initial development of synapses from preganglionic to postganglionic neurons is independent of signals derived from target tissues of postganglionic neurons [165] as is the formation of acetylcholine receptor clusters whose formation, however, is interrupted when preganglionic innervation is prevented [166]. Likewise in chick sympathetic ganglia acetylcholine receptor clustering and channel properties become altered during innervation by preganglionic fibers [167, 168]. In addition target- derived signals alter receptor composition and properties as exemplified by the effects of heart and kidney cells [169].

These studies outline aspects of synapse formation in a selected set of autonomic ganglia. They demonstrate induction of the genes coding for synaptic proteins before the onset of preganglionic and postganglionic contact formation. On the other hand they show the modulatory role of preganglionic innervation as well as target contact on the specification of the synaptic machinery. RNA sequencing analysis demonstrates enormous variation in transcript levels between neurons within sympathetic ganglia [83], which in part correlate with noradrenergic marker transcript levels. The significance of the variance with respect to different neuron subpopulations and activity states has to be clarified. In addition, the comparison of the synaptic protein expression profiles in sympathetic and parasympathetic neurons remains an open question.

#### Generation of parasympathetic postganglionic neurons from bi-potent glial precursor- like cells

Postganglionic parasympathetic neurons are generated later than sympathetic neurons and are located often at sites near their target tissues. The unexpected finding that these parasympathetic neurons are generated from glial progenitor-like cells associated with nerves [170, 171] explains both of these observations. The precursors migrate along with the outgrowing nerves to the periphery before they differentiate to neurons to form the ganglia. The analysis has been performed for cranial parasympathetic ganglia as well as heart ganglia [171] and pelvic ganglia [154, 170]. Similar to sympathetic precursors they express the transcription factor Sox10 characteristic for migrating neural crest cells and Phox2b expressed in all postganglionic autonomic neurons upon initiation of neuronal differentiation, yet different from these they express the glial marker PLP among others. Cell lineage analysis of these cells shows that they can differentiate to neurons as well as glial cells. Mutational inactivation of Ascl1 in mice prevents neuronal differentiation of the parasympathetic postganglionic neurons and diverts the cells to a glial fate. This differs from the situation in sympathetic ganglia where Ascl1 mutation results in the delayed but otherwise normal differentiation of the postganglionic neurons [172].

#### The pelvic ganglion displays a sympathetic not parasympathetic transcription factor fingerprint

Comparison of marker gene expression in autonomic postganglionic neurons of the mouse embryo from cranial to sacral levels discloses a similarity between cells in the paravertebral sympathetic chain and the pelvic ganglion and critical differences to those of cranial parasympathetic ganglia, strongly suggesting a homologous developmental origin of thoracolumbar and sacral postganglionic autonomic neurons [154]. While neurons in sympathetic and pelvic ganglia at different embryonic stages express the transcription factors Gata3 and Hand1, cranial parasympathetic neurons express Hmx2 and 3 (Table 2). Since Gata3 is critically required for the survival of embryonic sympathetic neurons as well as their maintenance in adult animals [173, 174] and absent from neurons in cranial parasympathetic ganglia [154], its expression is a strong indicator of the sympathetic lineage.

**Table 2:** Key regulators and their function in the development of the autonomic nervous system

Cranial	Thoracolumbar	Sacral
Preganglionic neurons		
Phox2b, Phox2a^a^	–	–
–	Olig2^b^	Olig2^c^
.	HoxC9^d^	nd
Tbx20^e^	–	–
–	FoxP1^f^	FoxP1^g^
–	Isl2^h^	nd
Postganglionic neurons		
–	Gata3^i^	Gata3^j^
–	Hand1^k^	Hand1^l^
Hand2^m^	Hand2^n^	nd
–	HoxB8^o^	nd
Hmx1^p^	Hmx1^q^	nd
Hmx2 and 3^r^	-^r^	-^r^

In this table transcriptional regulators are shown which are differentially expressed between preganglionic and postganglionic autonomic neurons at different levels of the rostrocaudal axis

^a^progenitor domain specific expression [228]; neuronal differentiation, subtype specific marker expression [89, 229]; cell cycle exit promotion, controls migration to mantle layer [229]; pan neuronal induction, synchronization of subtype specification; repression of Olig 2 expression [175], neurite outgrowth, neuronal migration [176], promotion of TBX 20 and TBX 2 expression [152]; requirement for preganglionic neuron development [154]

^b^progenitor domain specific expression; neuronal glial subtype choice [230, 231]; requirement for preganglionic neuron development [154]

^c^requirement for preganglonic neuron development [154]

^d^mRNA in embryonic mouse spinal cord caudal to T3; area of postmitotic cells [232]; protein in chick embryo; caudal brachial through thoracic; area of postmitotic cells [233]; expression in progenitors, differentiation requirement, neuronal migration [234]; axonal projection [235]; control of FoxP1 expression [235, 236]

^e^embryonic expression [154, 237]; cell migration [177]

^f^embryonic expression [154, 236]; segregation of motor neuron fates [236, 238]; axon projection [238]

^g^embryonic expression [154]

^h^expression in somatic motoneurons (*sm*) but not in visceromotor (*vm*) or branchiomotor (*bm*) neurons in the hindbrain [227, 239, 240]; sm and vm/bm neurons at hindbrain levels are derived from different progenitors, whereas both are derived from the same progenitor in the spinal cord [212]; transient Isl2 expression required for preganglionic neuron development [212]

^i^expression in embryonic mouse sympathetic ganglia [241]; differential expression in cranial parasympathetic versus sympathetic ganglia in mouse embryo [154]; lethality in mutant embryos, noradrenaline deficiency [242]; disturbed differentiation in mutant embryos [173, 225]; survival requirement in embryos and adult animals [174]

^j^expression in pelvic ganglion [154]

^k^expression in mouse SCG during embryonic, postnatal and adult stage, survival requirement, regulates TrkA expression [243]; embryonic expression in mouse sympathetic ganglia [154]

^l^expression in embryonic pelvic ganglion [154]

^m^Hand2 is expressed in the mouse sphenopalatine ganglion and is not connected to noradrenergic phenotype expression [147]; absence from majority of embryonic chick ciliary neurons [146, 148]

^n^expression in chick sympathetic ganglia [244];cross-regulation with Phox2b, noradrenergic induction [226]; Hand1 induction [223, 245]; Th and Dbh induction [150, 223, 224]; maintenance of Th and Dbh expression [149]

^o^expression in chick embryonic sympathetic ganglia, absence from embryonic chick ciliary ganglia, Hand2 induction in neural crest progenitors, Th and Dbh inductionin neural crest progenitors [148]

^p^transient expression in mouse ciliary ganglion [246]

^q^maintained expression in mouse sympathetic ganglia, required for TrkA expression, for Th maintenance, not for Vmat2 and Dbh [143]

^r^expression in mouse parasympathetic ganglia but not in mouse sympathetic and pelvic ganglia [154]

*nd* Not determined

Mutational inactivation of Olig2 required for motoneuron differentiation results in the lack of preganglionic nerves, which surprisingly did not affect the size of the pelvic ganglion [154]. In addition, glial- like precursor cells expressing Sox10 and PHOX2b could not be observed on the nerves traversing the splanchnic region surrounding the developing pelvic ganglion. As such, these authors conclude that the precursors forming this ganglion resemble those forming sympathetic ganglia and not those forming cranial parasympathetic ganglia. The discrepancy with the results of Sox10 lineage-tracing identifying pelvic ganglia as parasympathetic [170] remains to be resolved.

#### The transcriptional fingerprint of developing preganglionic neurons differs between cranial and all spinal levels

**Fig. 3:**
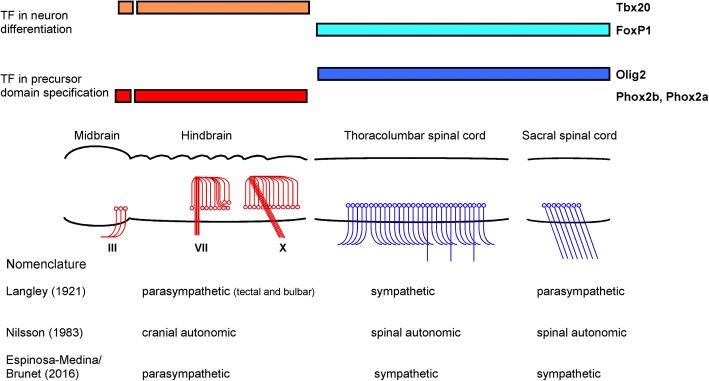
Preganglionic autonomic neurons, key transcription factors in their embryonic development and the classification of autonomic nervous system domains. The figure schematically displays the sympathetic and parasympathetic domains of autonomic preganglionic neurons and provides classical and recent naming proposals for the distinct subdivisions. These are derived primarily from physiological and pharmacological studies (Langley [1] see figure 1), evolutionary comparison within vertebrates (Nilsson [200], see figure 2) and developmental studies of critical regulators of neuronal cell lineage (Brunet and colleagues first presented in Espinosa–Medina et al., [154], see figure 4). The transcription factors responsible for the current renaming proposal from sacral “parasympathetic” to “sympathetic” are depicted above the schematic illustration of consecutive domains of the central nervous system harboring preganglionic autonomic neurons. The expression patterns and function are discussed in the main text and Table 2. The illustration is modified from Osumi and colleagues [227] with the mesencephalon containing the neuronal cell bodies giving rise to preganglionic axons to the IIIrd cranial nerve (N. oculomotorius) and the parasympathetic ciliary ganglion. The schematic illustration of the metencephalon displays rhombomeres 1 to 7 with the neuronal cell bodies giving rise to the visceromotor axons in the VIIth (N. facialis) and Xth (N. vagus) cranial nerves. The source of the Vth (N. trigeminus) and IXth nerve (N. glossopharyngeus) are omitted for simplicity. Within the thoracolumbar sympathetic domain, three different axon trajectories are indicated: leftward orientation indicating the rostral direction of preganglionic axons towards the SCG, rightward orientation towards more caudally located ganglia in the paravertebral sympathetic chains, and downward orientation indicating projection to prevertebral sympathetic ganglia. In the sacral autonomic domain at the right end of the scheme, axon projections indicate nerve fibers not entering the paravertebral sympathetic chain and traversing abdominal space in the N. pelvicus and pudentus

Analysis of marker genes at the sites of preganglionic neuron development in the mouse embryo demonstrates two distinct patterns of transcription factor expression at cranial as compared to thoracolumbar and sacral levels [154]. While Tbx2, 3 and 20 and Phox2b are expressed at the sites of cranial preganglionic neuron differentiation, they are absent not only at thoracolumbar but also sacral levels of preganglionic neuron development where FoxP1 is expressed instead [154] (Table 2; Fig. 3). Similar to the thoracolumbar region, preganglionic neuron development in the sacral domain depends on the transcription factor Olig2 which is also required for somatic motor neuron development [154]. Cranial preganglionic neuron development, different from thoracolumbar and sacral preganglionic neuronal differentiation, requires Phox2b [89]. In Phox2b mutant mice all cranial visceromotor as well as branchiomotor neurons are absent while somatic motoneurons at spinal levels are unaffected. Importantly, overexpression of Phox2b in the spinal cord of chick embryos results in neurite outgrowth and exit of the axon via the dorsal roots [175, 176]. Tbx2 and 20 expression depends on Phox2b [152]. For Tbx20 a function in the migration of cranial motoneurons during development has been demonstrated [177]. Thus the sequential action of Phox2b and Tbx20 appears to play critical roles in the differentiation, migration and axon projection characteristic of cranial motoneurons and distinct from spinal preganglionic motoneurons.

#### Apportionment of preganglionic to postganglionic synaptic connectivity

By electrophysiological recording and cell labeling techniques synaptic input from parasympathetic and sympathetic preganglionic neurons to individual postganglionic cells was quantified to study the features of ganglionic information processing. Analysis of the rodent submandibular ganglion [178, 179], the rabbit ciliary ganglion [180] and the hamster superior cervical ganglion [181] demonstrated a decrease of the number of preganglionic neurons innervating individual postganglionic cells during postnatal to adult stages.

During postnatal development the number of preganglionic fibers innervating individual neurons in the submandibular ganglion decreases while the number of synaptic boutons increases resulting in ganglion cells innervated by single preganglionic neurons in the rat [178]. Comparison of the submandibular ganglion in different mammalian species including rat, hamster, guinea pig and rabbit shows that the number of preganglionic axons terminating onto one postganglionic cell correlates with the dendritic complexity of the postganglionic cell and the size of the animal [182]. In the rabbit ciliary ganglion the number of preganglionic neurons innervating single postganglionic cells is postnatally reduced from four to two on average in adulthood [180]. As the cell number remains fairly constant and the counts of synaptic boutons increases during this period, the observations demonstrate the reorganization of preganglionic innervation focusing the input of single preganglionic cells to a reduced set of postganglionic cells. Interestingly, the input of one preganglionic neuron is confined to a restricted domain of the dendritic arbor of the postganglionic cell [183].

In the guinea pig superior cervical ganglion, a preganglionic fiber is estimated to contact 50 to 200 postganglionic neurons [184]. An individual postganglionic neuron receives a minimum of 10 preganglionic inputs derived on average from four adjacent thoracic spinal cord segments [185]. The numbers in the hamster superior cervical ganglion are somewhat smaller with an average 6 to 7 preganglionic fibers from 2 to 3 spinal cord segments converging onto each postganglionic neuron in the mature animal compared to more than 11 preganglionic fibers from 3 to 4 spinal cord segments in young specimens [181]. Depriving postganglionic neurons of the guinea pig superior cervical ganglion of their target reduces ganglionic transmission and the number of synaptic profiles, a process that can be prevented by nerve growth factor administration [121]. Application of NGF antiserum in guinea pigs results in the reduction of preganglionic transmission to and synaptic bouton numbers on superior cervical ganglion neurons [186] indicating that target – derived nerve growth factor is required to establish and maintain the normal synaptic complement of the cells.

Crucial progress came from NGF mutant mice where the loss of sympathetic neurons was prevented by the additional mutation of the proapoptotic gene Bax [187]. In newborn double mutant animals, the formation of pre- as well as postsynaptic specializations was severely compromised. Activated trkA endosomes retrogradely transported from the target within the axon via the cell soma towards the dendrites are required to form and maintain the normal synaptic bouton complement [122]. In addition, brain-derived neurotrophic factor (BDNF) produced by preganglionic neurons promotes synapse formation onto postganglionic neurons [188]. In BDNF mutant mice, the number of synapses onto superior cervical ganglion neurons in mature animals is reduced whereas BDNF overexpression with the help of the DBH promoter results in hyperinnervation and increases synapse number [188]. Importantly, neural activity affects the action of NGF and BDNF on synapse formation [189, 190] thereby pointing to a mechanism by which neurotrophins and nerve cell activity could regulate synapse formation from pre-to postganglionic sympathetic neurons in a quantitative manner. Furthermore growth factors from other families may be involved in this process as shown by conditional inactivation of the bone morphogenetic protein receptor BMPR1a in sympathetic ganglia of adult mice [191].

Thus, innervation and synapse formation in sympathetic ganglia are controlled by different families of growth factors, in particular neurotrophin signaling. The corresponding factors involved in synapse formation in parasympathetic ganglia are not resolved. In addition, the factors that regulate the establishment of specific connections in different autonomic pathways are unknown.

#### Developmental specification of the connections from preganglionic to postganglionic neurons and to target tissues – the unresolved domain

The question of how the specific connections from preganglionic neurons to the target tissue are brought about is unresolved. This entails a number of different problems of distinct interest. A very productive approach has been applied to somatic motoneuron development [192] where transcription factors required for motoneuron innervation of specific muscle targets have been characterized and their target genes specified. Such analysis is currently not available for the autonomic nervous system. Yet PHOX2b overexpression studies indicate that this transcription factor may be involved in the choice of dorsal axon exit in the hindbrain by parasympathetic cranial preganglionic neurons [176]. Preganglionic neurons in the spinal cord at bona fide sympathetic thoracolumbar levels as well as sacral levels choose ventral exit points similar to their developmentally related Olig2-dependent somatic motoneurons. The question here is, how the preganglionic axons fasciculate and then segregate from the ventral root as a white ramus. The preganglionic axons form the white ramus to enter the paravertebral sympathetic chain and synapse on postganglionic neurons in the paravertebral ganglia or to traverse and terminate in prevertebral ganglia, onto neurons of the artery-associated plexuses or the pelvic plexuses or ganglion. The molecular determinants of these decisions are currently unknown, which highlights the unresolved question of the formation of the white rami and the difference between cranial, thoracolumbar and sacral trajectories of preganglionic axons.

Molecular understanding is, however, emerging concerning the question of the formation of the paravertebral sympathetic strands and their projections. Neuropilin 1 and 2, receptors for semaphorins and vascular endothelial growth factor, are expressed in postganglionic sympathetic neurons [193]. Mutational inactivation of neuropilin 1 or semaphorin 3A results in disturbed migration of sympathetic neurons, aggregation to ganglia and fasciculation of their axons resulting in a misplacing of the neuronal cell bodies and the disturbance of the axon trunks forming the paravertebral ganglia chain [194]. In addition, semaphorin 3A and VEGF-levels regulate vascular innervation by sympathetic neurons [195] where VEGF reduces semaphorin 3A-evoked growth cone collapse [196]. In addition, other growth factors are involved in the regulation and direction of postganglionic sympathetic axon outgrowth. Endothelins, vascular endothelial derived factors, are critical for pathway selection of axons from superior cervical ganglion neurons on their way to targets in the head [197] and the heart [198]. Netrin 1, produced in vascular smooth muscle cells, and acting on the Dcc 1 (deleted in colorectal cancer) receptor expressed in sympathetic neurons is required for normal vascular innervation and vasoconstriction as shown in conditional mutant mice [199]. The full complement of the factors acting to position postganglionic sympathetic neurons at the ganglionic sites and to direct the outgoing axons to the precise targets are unknown but important first steps have been undertaken.

Two key unresolved problems are the transition from axon outgrowth to target innervation and the establishment, competition for and maintenance of synapses. These critical events during the development of a neural network composed of diverse target–specific pathways are incompletely understood for the autonomic nervous system. To what extent population specific cell surface markers may play a role in this process is still unknown. The critical issue of how the neural circuits to the different, in part closely associated autonomic targets such as sweat glands and the neighboring blood vessels are selectively innervated is still open for analysis.

#### Summarizing remarks

A range of cell biological, molecular and surgical techniques established a detailed knowledge of the development of the noradrenergic transmitter phenotype in postganglionic sympathetic neurons including the growth factor signaling systems and the transcription factors required for induction, differentiation and maintenance of the cells. The differentiation of the cholinergic postganglionic parasympathetic neurons is not characterized in its molecular mechanism. Yet, the transcription factors expressed in these neurons during development are known at least in part. This is also the case for the preganglionic neurons in both the sympathetic and parasympathetic system. With the increasing characterization of the transcription factors expressed and required in sympathetic and parasympathetic neurons, the close relation of thoracolumbar and sacral autonomic neurons and the difference from cranial autonomic neuronal development at both preganglionic and postganglionic levels is recognized and provokes the renaming of the sacral division of the autonomic nervous system as sympathetic.

With electrophysiological and histochemical techniques, subpopulations of postganglionic sympathetic neurons have been characterized and their target tissues described. RNA sequencing techniques are now complementing this approach to molecularly specify the efferent sympathetic outflow pathways. This approach is still lacking for the postganglionic parasympathetic neurons and the preganglionic neurons of both branches of the autonomic nervous system.

A major quest concerning the development of the autonomic nervous system remains the understanding of process outgrowth from both preganglionic and postganglionic neurons and the establishment of neuronal specificity. Some molecular players have been identified but the picture is far from complete. The question how the outgrowing neuronal processes are directed to and choose among alternative target structures and how the strength of the synaptic connections is established and regulated are key problems for the coming years.

## Conclusions

The classical scheme of the autonomic nervous system as delineated by Langley and Gaskell included a cranial and a sacral parasympathetic domain divided by a thoracolumbar sympathetic domain. One critical argument for the classical subdivision of the sympathetic and parasympathetic systems was the anatomical segregation along the body axes of the preganglionic outflow from the cranial, the thoracolumbar and the sacral level by gaps devoid of white communicating rami containing myelinated preganglionic fibers. In addition to the anatomical location of the preganglionic neuronal cell bodies, a range of arguments are brought forward which do not provide unequivocal criteria. These weaker arguments include the neurotransmitter phenotype, the distance from postganglionic cell bodies to target tissue and the opposite action of sympathetic and parasympathetic stimulation on a range of target organs. Yet, the classical subdivision of the domains in the autonomic nervous system is only partially supported by the molecular signatures specifying cellular differentiation.

### Is the sacral autonomic outflow parasympathetic or sympathetic?

With the analysis of the transcription factors expressed in the precursors and differentiating neurons of the cranial, thoracolumbar and sacral domains involved in the generation of preganglionic and postganglionic autonomic neurons [154], strong evidence has been obtained to indicate that thoracolumbar and sacral developmental pathways to autonomic neurons show critical similarities and differ fundamentally from cranial pathways. The critical argument rests on cell-autonomously acting developmental regulators, specifying neuronal progenitor domains, directing neuronal subtype differentiation and affecting the direction of axonal outgrowth, which are shared between the thoracolumbar and the sacral domain and are different from the cranial region (Fig. 3). This enforces the renaming of the sacral autonomic domain under the same label as the thoracolumbar domain. As the succession of transcription factor expression, i.e. the central regulators of cell type- specific gene expression, has been established as the gold standard for classification of cell populations and tissue types, these results provide a strong argument to rename the sacral autonomic neural pathways from parasympathetic to sympathetic.

Alternatively, the term spinal autonomic appears appropriate. Indeed, based on comparative anatomical analysis between vertebrate classes, the term “spinal autonomic” was proposed earlier to include the sympathetic and the sacral, then called parasympathetic, autonomic system [200] (Fig. 3). It remains to be seen, to what extent developmental and evolutionary analysis are going to converge and support the proposed renaming.

### Conflicting views on the naming of autonomic nervous system subdivisions

As already emphasized by Langley [1], the term “sympathetic” in Winslow‘s use included not only the intercostal nerve, the paravertebral sympathetic chain, but also the vagus nerve. On the other hand, Gaskell [7], page 15), was so impressed by the consensus between the anatomical paths, histological features and physiological action of the vaso-motor nerves, that he considered it more appropriate to replace the “meaningless title of main sympathetic chain” by “the chain of vaso-motor ganglia”. This idea, however, was abandoned. Another problem in subdividing the autonomic nervous system already recognized since Langley was the composition of the parasympathetic system including three subdivisions leading to the impression [201] that the term “parasympathetic” is a basket for everything being efferent but neither sympathetic nor somatic motoneuron targeting striated muscle.

The recent description of developmental regulators involved in the differentiation of autonomic neurons and the proposed renaming of the “sacral autonomic outflow” initiated a heated dispute [202–208]. The groups defending the classical naming [202–205, 208] correctly point to the importance of the description of diverse autonomic pathways to the different target organs subserving the complex homeostatic integration characteristic of the autonomic nervous system. Yet, they are not able to convincingly defend why the proposed renaming of the sacral autonomic domain would conflict with the appreciation of this autonomic complexity. In addition, they vaguely refer to pharmacological and tutorial arguments to maintain the classical nomenclature. Neither of the latter two categories of arguments is strong, as realized already by Langley in his work on the pharmacology of sweat secretion and much more by his discussion of the terms “autonomic”, “involuntary”, “vegetative” and, most importantly, the term “sympathetic”.

From the rebuttal discussions [202, 208, 209] it seems that transcription factor profiles are seen merely as embryonic signatures not intimately related to the mature physiological standing of the neuron under consideration. In particular, the importance to understand the development of the different autonomic neuron populations in the diverse sympathetic and parasympathetic pathways to different targets is not evident from these discussions. This view neglects the evidence obtained in diverse neuronal populations from different animal phyla demonstrating the linkage of transcriptional control of neuron subtype development and the acquisition as well as maintenance of subtype–specific functional properties of the neuronal elements constituting the diverse neural circuits [144, 210, 211].

The visceral motoneurons at hindbrain level are derived from different progenitor populations than the somatic motoneurons whereas both are derived from the same progenitor population in the spinal cord [212]. This is reflected in the highly divergent profile of transcription factors essential for their differentiation (Fig. 3; Table 2). The identity of critical factors, in particular Olig2 and FoxP1 as compared to Phox2B and TBX20, along the rostrocaudal axis of the spinal cord from thoracolumbar to sacral levels poses no trivial fact as suggested [209] but reflects a basic developmental program realized in vertebrates. This program organizes spinal as compared to hindbrain nervous tissue, and sets up the position, identity and axonal projection of neurons, which are prerequisites for, rather than consequences of their physiological function (compare [205]). It is within this “spinal” developmental, anatomical and connectivity framework that we understand the sympathetic autonomic outflow. Recent studies into molecular development demonstrate that critical characteristics of this framework also apply to the sacral autonomic region and are fundamentally different from those of the cranial autonomic domain.

### Future results of interest that may affect the naming choices for autonomic nervous system subdivisions

The amazing progress with RNA sequencing techniques that allow quantitative detection of message transcribed from each gene within the cellular genome provided a quantum leap in the characterization of gene expression patterns within cell populations and single cells. Among autonomic neurons detailed data are available for postganglionic sympathetic neurons derived from stellate and thoracic ganglia [80, 83]. Genome wide expression data for cranial, heart and abdominal autonomic ganglia can be expected and will provide invaluable insight into the diversification of sympathetic and parasympathetic neurons. Of particular interest will be the characterization of the subpopulations in the cardiac ganglia and the pelvic ganglia as both contain populations of cholinergic and noradrenergic cells. The comparison of data from pelvic and celiac ganglion neurons versus superior cervical and stellate ganglion neurons will be highly interesting as it can be expected to show differences between postganglionic neurons distinguished by their migration and final settlement with respect to the distance to the spinal cord and their origin in the neural crest. Finally, comparison with the transcriptomes of the neurons in the cranial parasympathetic ganglia will answer a range of questions including the difference between primarily noradrenergic sympathetic and cholinergic parasympathetic neurons.

More challenging but at least as important will be the characterization by RNA sequencing of preganglionic sympathetic and parasympathetic neurons at all levels of the body axis. Again a host of questions will be addressed and the comparison to somatic motoneurons on the one hand and the subpopulations of preganglionic autonomic neurons on the other hand can be expected to provide crucial progress. A key problem linked to this approach is the molecular understanding of the pathways taken by preganglionic neurons from either system. Aspects of this topic are the understanding of the differences between preganglionic sympathetic neurons targeting postganglionic neurons in the paravertebral sympathetic chain, those targeting neurons in the prevertebral ganglia and those destined to innervate more distal plexuses. Another crucial point is the understanding of the molecular control of target innervation in cases where the sympathetic and the parasympathetic system innervate different sites within the same target tissue, i.e. the ciliary body, the heart and the pelvic organs.

### Bringing about the sympathies of the body

Already some 300 years ago the sympathetic paravertebral chain and vagus nerve, then called the great and the medium sympathetic ([3], partially translated by Langley [1]), were considered to mediate the functional harmony between the organs. With critical advances in physiological and pharmacological investigation some 100 years ago, these two domains of the nervous system, the sympathetic and parasympathetic autonomic branches, became appreciated as often working antagonistically and operating with different transmitter systems to balance the working of many organs [1, 5]. The fusion of this line of thought with considerations of the homeostasis of bodily functions in response to different stressor or relaxation settings [4] resulted in the appealing yet too often oversimplified thinking of two antagonistic efferent systems ensuring balance of key physiological parameters from “fight and flight” to “rest and digest” situations (see [201], for critical discussion).

The molecular and physiological characterization of the autonomic neurons has provided very refined knowledge of neuron subpopulations distinguished by their neurochemistry, electrical activity and reflex behavior upon sensory stimulation. The integration into autonomic networks is only partially understood, however. The molecular identity of the preganglionic and postganglionic neurons synaptically linked in distinct autonomic pathways is still undefined at the level of the molecular players mediating the specific synaptic connection. In addition, the postganglionic sympathetic neurons targeting major organs such as heart, lung and kidney are not yet characterized by their full transcriptional fingerprint. Thus, a significant gap remains between the cellular characterization of autonomic neurons and the understanding of their embedding in neural networks mediating homeostatic control. Two key systems coordinating organ functions, the cardio – respiratory balancing of respiration and perfusion [213, 214] and the cardio – renal control of the fluid matrix [215–217] are directed by autonomic neurons whose molecular fingerprints are not resolved to an extent that allows their distinction from other autonomic pathways during development or in the adult organism.

Taken together, the molecular and developmental characterization of selected autonomic neuron subgroups [80] awaits to be extended to those physiologically and anatomically defined neurons supplying key organs involved in homeostatic regulatory processes such as heart, lung, kidney as well as skin, gut and bladder. A major open question is how the appropriate pairs of preganglionic and postganglionic partners connect during development to establish the diverse and distinct neural pathways regulating different target tissues [218–220]. An even greater challenge is the central connection of the preganglionic neurons and their full embedment into reflex arcs starting with sensory neurons.

The synthesis of physiological characterization and molecular identification in combination with developmental analysis of autonomic neurons promises not only a comprehensive understanding of the neural networks underlying homeostatic regulation of the body functions but also of their emergence during vertebrate development and evolution.

## Abbreviations

Ascl1: Achaete- scute family bHLH transcription factor 1
Bax: BCL2 associated X, apoptosis regulator
BDNF: Brain-derived neurotrophic factor
BMP: Bone morphogenetic protein
ChAT: Choline acetyltransferase
DBH: Dopaminebeta-hydroxylase
DDC: Deleted in colorectal carcinoma
FoxP1: Forkhead box P1
Gata2/3: GATA binding protein 2/3
GDNF: Glial cell line derived neurotrophic factor
Hand1/2: Heart and neural crest derivatives expressed1/2
Hb9 (Mnx1): Motor neuron and pancreas homeobox 1
Hmx1: H6 homeobox1
Hoxb8: Homeobox b8
HRP: Horseraddish peroxidase
Islet1/2: ISL LIM homeobox1/2
NGF: Nerve growth factor
Olig2: Oligodendrocyte transcription factor 2
Phox2a/b: Paired-like homeobox 2a/b
PLP: Proteolipid protein
Ret: Ret proto-oncogene
Sox10: SRY-box 10
Tbx: T-box transcription factor
Th: Tyrosine hydroxylase
trkA: (Ntrk1) neurotrophic tyrosine kinase receptor 1
VAChT: Vesicular acetylcholine transporter
VEGF: Vascular endothelial growth factor

## References

[1] Langley JN. The autonomic nervous system (Pt 1). 1921. W. Heffer & Sons Ltd. Cambridge

[2] Langley JN (1916). Sketch of the progress of discovery in the eighteenth century as regards the autonomic nervous system. J Physiol.

[3] Winslow JB (1732). Exposition anatomique de la structure du corps humain.

[4] Cannon WB (1963). The wisdom of the body: Norton library.

[5] Gaskell WH (1920). The involuntary nervous system.

[6] Gaskell WH (1880). On the tonicity of the heart and blood vessels. J Physiol.

[7] Gaskell WH (1886). On the structure, distribution and function of the nerves which innervate the visceral and vascular systems. J Physiol.

[8] Gaskell WH (1889). On the relation between the structure, function, distribution and origin of the cranial nerves; together with a theory of the origin of the nervous system of vertebrata. J Physiol.

[9] Langley JN, Anderson HK (1895). The innervation of the pelvic and adjoining viscera: part II. The bladder. Part III. The external generative organs. Part IV. The internal generative organs. Part V. Position of the nerve cells on the course of the efferent nerve Fibres. J Physiol.

[10] Langley JN, Anderson HK (1895). On the innervation of the pelvic and adjoining viscera: part I. The lower portion of the intestine. J Physiol.

[11] Langley JN, Anderson HK (1896). The innervation of the pelvic and adjoining viscera: part VII. Anatomical observations. J Physiol.

[12] Langley JN, Anderson HK (1896). The innervation of the pelvic and adjoining viscera: part VI. Histological and physiological observations upon the effects of section of the sacral nerves. J Physiol.

[13] Langley JN, Uyeno K (1922). The secretion of sweat: part II. The effect of vaso-constriction and of adrenaline. J Physiol.

[14] Langley JN (1905). On the reaction of cells and of nerve-endings to certain poisons, chiefly as regards the reaction of striated muscle to nicotine and to curari. J Physiol.

[15] Pick J, Sheehan D (1946). Sympathetic rami in man. J Anat.

[16] Kuntz A (1945). The autonomic nervous system.

[17] Jänig W (2006). The integrative action of the autonomic nervous system: neurobiology of homeostasis.

[18] von Bezold A (1863). Untersuchungen über die Innervation des Herzens.

[19] Piccolino M, Bresadola M (2013). Shocking Fraogs: Galvani, Volta, and the electric origins of neuroscience.

[20] Bayliss WM, Starling EH (1892). On some points in the innervation of the mammalian heart. J Physiol.

[21] Samaan A (1935). The antagonistic cardiac nerves and heart rate. J Physiol.

[22] Samaan A (1935). Muscular work in dogs submitted to different conditions of cardiac and splanchnic innervations. J Physiol.

[23] Rosenblueth A, Simeone FA (1934). The interrelations of vagal and accelerator effects on the cardiac rate. Am J Physiol.

[24] Levy MN (1971). Sympathetic-parasympathetic interactions in the heart. Circ Res.

[25] Randall WC (1977). Neural regulation of the heart.

[26] Randall WC (1984). Nervous control of cardiovascular function.

[27] Armour JA, Ardell JL (1994). Neurocardiology.

[28] Koizumi K, Terui N, Kollai M (1983). Neural control of the heart: significance of double innervation re-examined. J Auton Nerv Syst.

[29] Ernst G (2017). Heart-rate variability-more than heart beats?. Front Public Health.

[30] Mizeres NJ (1957). The course of the left cardioinhibitory fibers in the dog. Anat Rec.

[31] Mizeres NJ (1958). The origin and course of the cardioaccelerator fibers in the dog. Anat Rec.

[32] Randall WC, Mc NH, Cowan J, Caliguiri L, Rohse WG (1957). Functional analysis of the cardioaugmentor and cardioaccelerator pathways in the dog. Am J Phys.

[33] Randall WC, Milosavljevic M, Wurster RD, Geis GS, Ardell JL (1986). Selective vagal innervation of the heart. Ann Clin Lab Sci.

[34] Randall WC, Ardell JL, Wurster RD, Milosavljevic M (1987). Vagal postganglionic innervation of the canine sinoatrial node. J Auton Nerv Syst.

[35] Randall WC, Ardell JL, O'Toole MF, Wurster RD (1988). Differential autonomic control of SAN and AVN regions of the canine heart: structure and function. Prog Clin Biol Res.

[36] Coote JH (2013). Myths and realities of the cardiac vagus. J Physiol.

[37] Koizumi K, Brooks CM (1972). The integration of autonomic system reactions: a discussion of autonomic reflexes, their control and their association with somatic reactions. Ergeb Physiol Biol Chem Exp Pharmakol.

[38] Koizumi K, Kollai M (1992). Multiple modes of operation of cardiac autonomic control: development of the ideas from Cannon and brooks to the present. J Auton Nerv Syst.

[39] Kollai M, Koizumi K (1979). Reciprocal and non-reciprocal action of the vagal and sympathetic nerves innervating the heart. J Auton Nerv Syst.

[40] Kollai M, Koizumi K (1980). Patterns of single unit activity in sympathetic postganglionic nerves. J Auton Nerv Syst.

[41] Szulczyk P, Tafil M (1980). Influence of the input from left and right arterial baroreceptors on left inferior cardiac nerve activity in cats. J Auton Nerv Syst.

[42] Koizumi K, Seller H, Kaufman A, Brooks CM (1971). Pattern of sympathetic discharges and their relation to baroreceptor and respiratory activities. Brain Res.

[43] Koizumi K, Nishino H, Brooks CM (1977). Centers involved in the autonomic reflex reactions originating from stretching of the atria. Proc Natl Acad Sci U S A.

[44] Kollai M, Koizumi K, Yamashita H, Brooks CM (1978). Study of cardiac sympathetic and vagal efferent activity during reflex responses produced by stretch of the atria. Brain Res.

[45] Kollai M, Koizumi K (1977). Differential responses in sympathetic outflow evoked by chemoreceptor activation. Brain Res.

[46] Kollai M, Koizumi K, Brooks CM (1978). Nature of differential sympathetic discharges in chemoreceptor reflexes. Proc Natl Acad Sci U S A.

[47] Paton JF, Boscan P, Pickering AE, Nalivaiko E (2005). The yin and yang of cardiac autonomic control: Vago-sympathetic interactions revisited. Brain Res Brain Res Rev.

[48] Paton JF, Nalivaiko E, Boscan P, Pickering AE (2006). Reflexly evoked coactivation of cardiac vagal and sympathetic motor outflows: observations and functional implications. Clin Exp Pharmacol Physiol.

[49] Ribeiro JP, Ibanez JM, Stein R (1991). Autonomic nervous control of the heart rate response to dynamic incremental exercise: evaluation of the Rosenblueth-Simeone model. Eur J Appl Physiol Occup Physiol.

[50] Bootsma M, Swenne CA, Van Bolhuis HH, Chang PC, Cats VM, Bruschke AV (1994). Heart rate and heart rate variability as indexes of sympathovagal balance. Am J Phys.

[51] White DW, Raven PB (2014). Autonomic neural control of heart rate during dynamic exercise: revisited. J Physiol.

[52] Florea VG, Cohn JN (2014). The autonomic nervous system and heart failure. Circ Res.

[53] Shen MJ, Zipes DP (2014). Role of the autonomic nervous system in modulating cardiac arrhythmias. Circ Res.

[54] Olivas A, Gardner RT, Wang L, Ripplinger CM, Woodward WR, Habecker BA (2016). Myocardial infarction causes transient cholinergic Transdifferentiation of cardiac sympathetic nerves via gp130. J Neurosci.

[55] Coote JH, Chauhan RA (2016). The sympathetic innervation of the heart: important new insights. Auton Neurosci.

[56] Kawashima T (2005). The autonomic nervous system of the human heart with special reference to its origin, course, and peripheral distribution. Anat Embryol (Berl).

[57] Chuang KS, Liu WC, Liou NH, Liu JC (2004). Horseradish peroxidase localization of sympathetic postganglionic and parasympathetic preganglionic neurons innervating the monkey heart. Chin J Physiol.

[58] Kalia M (1981). Brain stem localization of vagal preganglionic neurons. J Auton Nerv Syst.

[59] Stuesse SL (1982). Origins of cardiac vagal preganglionic fibers: a retrograde transport study. Brain Res.

[60] Hopkins DA, Armour JA (1982). Medullary cells of origin of physiologically identified cardiac nerves in the dog. Brain Res Bull.

[61] Hopkins DA, Armour JA (1984). Localization of sympathetic postganglionic and parasympathetic preganglionic neurons which innervate different regions of the dog heart. J Comp Neurol.

[62] Shih CJ, Chuang KS, Tsai SH, Liu JC (1985). Horseradish peroxidase localization of the sympathetic postganglionic neurons innervating the cat heart. J Auton Nerv Syst.

[63] Purves D, Lichtman JW (1978). Formation and maintenance of synaptic connections in autonomic ganglia. Physiol Rev.

[64] Nja A, Purves D (1977). Specific innervation of guinea-pig superior cervical ganglion cells by preganglionic fibres arising from different levels of the spinal cord. J Physiol (London).

[65] McLachlan EM, Meckler RL (1989). Characteristics of synaptic input to three classes of sympathetic neurone in the coeliac ganglion of the guinea-pig. J Physiol.

[66] Janig W, Schmidt RF (1970). Single unit responses in the cervical sympathetic trunk upon somatic nerve stimulation. Pflugers Arch: Eur J Physiol.

[67] McLachlan EM, Hirst GD (1980). Some properties of preganglionic neurons in upper thoracic spinal cord of the cat. J Neurophysiol.

[68] Boczek-Funcke A, Dembowsky K, Habler HJ, Janig W, McAllen RM, Michaelis M (1992). Classification of preganglionic neurones projecting into the cat cervical sympathetic trunk. J Physiol.

[69] Horeyseck G, Janig W (1974). Reflexes in postganglionic fibres within skin and muscle nerves after mechanical non-noxious stimulation of skin. Exp Brain Res.

[70] Horeyseck G, Janig W (1974). Reflexes in postganglionic fibres within skin and muscle nerves after noxious stimulation of skin. Exp Brain Res.

[71] Janig W, Kummel H (1981). Organization of the sympathetic innervation supplying the hairless skin of the cat's paw. J Auton Nerv Syst.

[72] Janig W, McLachlan EM (1992). Characteristics of function-specific pathways in the sympathetic nervous system. Trends Neurosci.

[73] Janig W, Habler HJ (2000). Specificity in the organization of the autonomic nervous system: a basis for precise neural regulation of homeostatic and protective body functions. Prog Brain Res.

[74] Jobling P, Gibbins IL (1999). Electrophysiological and morphological diversity of mouse sympathetic neurons. J Neurophysiol.

[75] Janig W, Habler HJ (2003). Neurophysiological analysis of target-related sympathetic pathways--from animal to human: similarities and differences. Acta Physiol Scand.

[76] Ernsberger U, Rohrer H (1996). The development of the noradrenergic transmitter phenotype in postganglionic sympathetic neurons. Neurochem Res.

[77] Anderson RL, Morris JL, Gibbins LL (2001). Neurochemical differentiation of functionally distinct populations of autonomic neurons. J Comp Neurol.

[78] Anderson RL, Jobling P, Gibbins IL (2001). Development of electrophysiological and morphological diversity in autonomic neurons. J Neurophysiol.

[79] Lundberg JM, Hökfelt T, Änggard A, Terenius L, Elde R, Markey K, Goldstein M, Kimmel J (1982). Organizational principles in the peripheral sympathetic nervous system: subdivision by coexisting peptides (somatostatin-, avian pancreatic polypeptide-, and vasoactive intestinal polypeptide- like immunoreactive materials). Proc Natl Acad Sci U S A.

[80] Furlan A, La Manno G, Lubke M, Haring M, Abdo H, Hochgerner H, Kupari J, Usoskin D, Airaksinen MS, Oliver G (2016). Visceral motor neuron diversity delineates a cellular basis for nipple- and pilo-erection muscle control. Nat Neurosci.

[81] Enemar A, Falck B, Hakanson R (1965). Observations on the appearance of norepinephrine in the sympathetic nervous system of the chick embryo. Dev Biol.

[82] Ernsberger U, Reissmann E, Mason I, Rohrer H (2000). The expression of dopamine b-hydroxylase, tyrosine hydroxylase, and Phox2 transcription factors in sympathetic neurons: evidence for common regulation during noradrenergic induction and diverging regulation later in development. Mech Dev.

[83] Ernsberger U, Kramer M, Tsarovina K, Deller T, Rohrer H (2017). Coordinate expression of pan-neuronal and functional signature genes in sympathetic neurons. Cell Tissue Res.

[84] Elfvin L-G, Lindh B, Hökfelt T (1993). The chemical neuroanatomy of sympathetic ganglia. Annu Rev Neurosci.

[85] Francis NJ, Landis SC (1999). Cellular and molecular determinants of sympathetic neuron development. Ann Rev Neurosci.

[86] Schotzinger RJ, Landis SC (1990). Acquisition of cholinergic and peptidergic properties by sympathetic innervation of rat sweat glands requires interaction with normal target. Neuron.

[87] Stanke M, Duong CV, Pape M, Geissen M, Burbach G, Deller T, Gascan H, Otto C, Parlato R, Schütz G (2006). Target-dependent specification of the neurotransmitter phenotype: cholinergic differentiation of sympathetic neurons is mediated in vivo by gp130 signaling. Development (Cambridge, England).

[88] Hasan W (2013). Autonomic cardiac innervation: development and adult plasticity. Organogenesis.

[89] Pattyn A, Hirsch MR, Goridis C, Brunet JF (2000). Control of hindbrain motor neuron differentiation by the homeobox gene Phox2b. Development (Cambridge, England).

[90] Shirasaki R, Pfaff SL (2002). Transcriptional codes and the control of neuronal identity. Annu Rev Neurosci.

[91] Alaynick WA, Jessell TM, Pfaff SL (2011). SnapShot: spinal cord development. Cell.

[92] Nielsen KC, Owman C, Santini M (1969). Anastomosing adrenergic nerves from the sympathetic trunk to the vagus at the cervical level in the cat. Brain Res.

[93] Lundberg JM, Dahlstrom A, Larsson I, Petterson G, Ahlman H, Kewenter J (1978). Efferent innervation of the small intestine by adrenergic neurons from the cervical sympathetic and stellate ganglia, studied by retrograde transport of peroxidase. Acta Physiol Scand.

[94] Baron R, Janig W, McLachlan EM (1985). The afferent and sympathetic components of the lumbar spinal outflow to the colon and pelvic organs in the cat. III. The colonic nerves, incorporating an analysis of all components of the lumbar prevertebral outflow. J Comp Neurol.

[95] Baron R, Janig W, McLachlan EM (1985). The afferent and sympathetic components of the lumbar spinal outflow to the colon and pelvic organs in the cat. II. The lumbar splanchnic nerves. J Comp Neurol.

[96] Baron R, Janig W, McLachlan EM (1985). The afferent and sympathetic components of the lumbar spinal outflow to the colon and pelvic organs in the cat. I. The hypogastric nerve. J Comp Neurol.

[97] Janig W, McLachlan EM (1987). Organization of lumbar spinal outflow to distal colon and pelvic organs. Physiol Rev.

[98] de Groat WC, Yoshimura N (2001). Pharmacology of the lower urinary tract. Annu Rev Pharmacol Toxicol.

[99] de Groat WC, Griffiths D, Yoshimura N (2015). Neural control of the lower urinary tract. Compr Physiol.

[100] Nadelhaft I, Degroat WC, Morgan C (1980). Location and morphology of parasympathetic preganglionic neurons in the sacral spinal cord of the cat revealed by retrograde axonal transport of horseradish peroxidase. J Comp Neurol.

[101] Morgan C, Nadelhaft I, de Groat WC (1981). The distribution of visceral primary afferents from the pelvic nerve to Lissauer's tract and the spinal gray matter and its relationship to the sacral parasympathetic nucleus. J Comp Neurol.

[102] Nadelhaft I, Roppolo J, Morgan C, de Groat WC (1983). Parasympathetic preganglionic neurons and visceral primary afferents in monkey sacral spinal cord revealed following application of horseradish peroxidase to pelvic nerve. J Comp Neurol.

[103] Roppolo JR, Nadelhaft I, de Groat WC (1985). The organization of pudendal motoneurons and primary afferent projections in the spinal cord of the rhesus monkey revealed by horseradish peroxidase. J Comp Neurol.

[104] McKenna KE, Nadelhaft I (1986). The organization of the pudendal nerve in the male and female rat. J Comp Neurol.

[105] Cassell JF, Clark AL, McLachlan EM (1986). Characteristics of phasic and tonic sympathetic ganglion cells of the guinea-pig. J Physiol.

[106] Keast JR, McLachlan EM, Meckler RL (1993). Relation between electrophysiological class and neuropeptide content of guinea pig sympathetic prevertebral neurons. J Neurophysiol.

[107] Gibbins IL, Jobling P, Morris JL (2003). Functional organization of peripheral vasomotor pathways. Acta Physiol Scand.

[108] Keast JR (1999). Unusual autonomic ganglia: connections, chemistry, and plasticity of pelvic ganglia. Int RevCytol.

[109] Keast JR, de Groat WC (1989). Immunohistochemical characterization of pelvic neurons which project to the bladder, colon, or penis in rats. J Comp Neurol.

[110] Keast JR (1995). Visualization and immunohistochemical characterization of sympathetic and parasympathetic neurons in the male rat major pelvic ganglion. Neuroscience.

[111] Jobling P, Lim R (2008). Anatomical and physiological properties of pelvic ganglion neurons in female mice. Auton Neurosci.

[112] Levi-Montalcini R, Hamburger V (1953). A diffusible agent of mouse sarcoma producing hyperplasia of sympathetic ganglia and hyperneurotization of viscera in the chick embryo. J Exp Zool.

[113] Levi-Montalcini R, Booker B (1960). Destruction of the sympathetic ganglia in mammals by an antiserum to a nerve-growth protein. Proc Natl Acad Sci U S A.

[114] Levi-Montalcini R, Booker B (1960). Excessive growth of the sympathetic ganglia evoked by a protein isolated from mouse salivary glands. Proc Natl Acad Sci U S A.

[115] Edgar D, Barde Y-A, Thoenen H (1981). Subpopulations of cultured chick sympathetic neurones differ in their requirements for survival factors. Nature (London).

[116] Crowley C, Spencer SD, Nishimura MC, Chen KS, Pitts-Meek S, Armanini MP, Ling LH, McMahon SB, Shelton DL, Levinson AD (1994). Mice lacking nerve growth factor display perinatal loss of sensory and sympathetic neurons yet develop basal forebrain cholinergic neurons. Cell.

[117] Smeyne RJ, Klein R, Schnapp A, Long LK, Bryant S, Lewin A, Lira SA, Barbacid M (1994). Severe sensory and sympathetic neuropathies in mice carrying a disrupted Trk/NGF receptor gene. Nature.

[118] Fagan AM, Zhang H, Landis S, Smeyne RJ, Silos-Santiago I, Barbacid M (1996). TrkA, but not TrkC, receptors are essential for survival of sympathetic neurons *in vivo*. J Neurosci.

[119] Glebova NO, Ginty DD (2004). Heterogeneous requirement of NGF for sympathetic target innervation *in vivo*. J Neurosci.

[120] Ruit KG, Snider WD (1991). Administration or deprivation of nerve growth factor during development permanently alters neuronal geometry. J Comp Neurol.

[121] Nja A, Purves D (1978). The effects of nerve growth factor and its antiserum on synapses in the superior cervical ganglion of the guinea-pig. J Physiol.

[122] Lehigh KM, West KM, Ginty DD (2017). Retrogradely transported TrkA endosomes signal locally within dendrites to maintain sympathetic neuron synapses. Cell Rep.

[123] Falck B, Hilarp NA, Theme G, Torp A (1962). Fluorescence of catecholamines and related compounds with formaldehyde. J Histochem Cytochem.

[124] Kirby ML, Gilmore SA (1976). A correlative histofluorescence and light microscopic study of the formation of the sympathetic trunks in chick embryos. Anat Rec.

[125] Allan IJ, Newgreen DF (1977). Catecholamine accumulation in neural crest cells and the primary sympathetic chain. Am J Anat.

[126] Fairman K, Giacobini E, Chippinelli V (1976). Developmental variations of tyrosine hydroxylase and acetylcholinesterase in embryonic and post hatching chicken sympathetic ganglia. Brain Res.

[127] Cochard P, Goldstein M, Black IB (1978). Ontogenetic appearance and disappearance of tyrosine hydroxylase and catecholamines in the rat embryo. Proc Natl Acad Sci U S A.

[128] Cochard P, Goldstein M, Black IB (1979). Initial development of the noradrenergic phenotype in autonomic neuroblasts of the rat embryo in vivo. Dev Biol.

[129] Teitelman G, Baker H, Joh TH, Reis DJ (1979). Appearance of catecholamine-synthesizing enzymes during development of rat sympathetic nervous system:possible role of tissue environment. Proc Natl Acad Sci U S A.

[130] Coughlin MD, Boyer DM, Black IB (1977). Embryonic development of a mouse sympathetic ganglion in vivo and in vitro. Proc Natl Acad Sci U S A.

[131] Le Douarin NM, Smith J, Le Lievre CS (1981). From the neural crest to the ganglia of the peripheral nervous system. Annu Rev Physiol.

[132] Le Douarin NM, Teillet MA (1974). Experimental analysis of the migration and differentiation of neuroblasts of the autonomic nervous system and of neurectodermal mesenchymal derivatives, using a biological cell marking technique. Dev Biol.

[133] Espinosa-Medina I, Jevans B, Boismoreau F, Chettouh Z, Enomoto H, Muller T, Birchmeier C, Burns AJ, Brunet JF (2017). Dual origin of enteric neurons in vagal Schwann cell precursors and the sympathetic neural crest. Proc Natl Acad Sci U S A.

[134] Cohen A, Fuxe K, Olson L, Zotterman Y (1974). DNA synthesis and cell division in differentiating avian adrenergic neuroblasts. Wenner-Gren Center International Symposion Series.

[135] Rothman TP, Gershon MD, Holtzer H (1978). The relationship of cell division to the acquisition of adrenergic characteristics by developing sympathetic ganglion cell precursors. Dev Biol.

[136] Rohrer H, Thoenen H (1987). Relationship between differentiation and terminal mitosis: chick sensory and ciliary neurons differentiate after terminal mitosis of precursor cells whereas sympathetic neurons continue to divide after differentiation. J Neurosci.

[137] Gonsalvez DG, Cane KN, Landman KA, Enomoto H, Young HM, Anderson CR (2013). Proliferation and cell cycle dynamics in the developing stellate ganglion. J Neurosci.

[138] Holzmann J, Hennchen M, Rohrer H (2015). Prox1 identifies proliferating neuroblasts and nascent neurons during neurogenesis in sympathetic ganglia. Dev Neurobiol.

[139] Ernsberger U, Patzke H, Tissier-Seta JP, Reh T, Goridis C, Rohrer H (1995). The expression of tyrosine hydroxylase and the transcription factors cPhox-2 and Cash-1: evidence for distinct inductive steps in the differentiation of chick sympathetic precursor cells. Mech Dev.

[140] Pattyn A, Morin X, Cremer H, Goridis C, Brunet J-F (1999). The homeobox gene Phox2b is essential for the development of all autonomic derivatives of the neural crest. Nature.

[141] Ernsberger U, Patzke H, Rohrer H (1997). The developmental expression of choline acetyltransferase (ChAT) and the neuropeptide VIP in chick sympathetic neurons: evidence for different regulatory events in cholinergic differentiation. Mech Dev.

[142] Huber K, Ernsberger U (2006). Cholinergic differentiation occurs early in mouse sympathetic neurons and requires Phox2b. Gene Expr.

[143] Furlan A, Lubke M, Adameyko I, Lallemend F, Ernfors P (2013). The transcription factor Hmx1 and growth factor receptor activities control sympathetic neurons diversification. EMBO J.

[144] Rohrer H (2011). Transcriptional control of differentiation and neurogenesis in autonomic ganglia. Eur J Neurosci.

[145] Chan WH, Anderson CR, Gonsalvez DG (2018). From proliferation to target innervation: signaling molecules that direct sympathetic nervous system development. Cell Tissue Res.

[146] Müller F, Rohrer H (2002). Molecular control of ciliary neuron development: BMPs and downstream transcriptional control in the parasympathetic lineage. Development (Cambridge, England).

[147] Stanzel S, Stubbusch J, Pataskar A, Howard MJ, Deller T, Ernsberger U, Tiwari VK, Rohrer H, Tsarovina K (2016). Distinct roles of Hand2 in developing and adult autonomic neurons. Dev Neurobiol.

[148] Huber L, Ferdin M, Holzmann J, Stubbusch J, Rohrer H (2012). HoxB8 in noradrenergic specification and differentiation of the autonomic nervous system. Dev Biol.

[149] Schmidt M, Lin S, Pape M, Ernsberger U, Stanke M, Kobayashi K, Howard MJ, Rohrer H (2009). The bHLH transcription factor Hand2 is essential for the maintenance of noradrenergic properties in differentiated sympathetic neurons. Dev Biol.

[150] Lucas ME, Müller F, Rüdiger R, Henion PD, Rohrer H (2006). The bHLH transcription factor hand2 is essential for noradrenergic differetiation of sympathetic neurons. Development (Cambridge, England).

[151] Burau K, Stenull I, Huber K, Misawa H, Berse B, Unsicker K (2004). Ernsberger U: c-ret regulates cholinergic properties in mouse sympathetic neurons: evidence from mutant mice. Eur J Neurosci.

[152] Coppola E, d’Autreaux F, Rijli FM, Brunet JF (2010). Ongoing roles of Phox2 homeodomain transcription factors during neuronal differentiation. Development (Cambridge, England).

[153] Ernsberger U, Rohrer H (1999). Development of the cholinergic neurotransmitter phenotype in postganglionic sympathetic neurons. Cell Tissue Res.

[154] Espinosa-Medina I, Saha O, Boismoreau F, Chettouh Z, Rossi F, Richardson WD, Brunet JF (2016). The sacral autonomic outflow is sympathetic. Science.

[155] Leblanc GG, Landis SC (1989). Differentiation of noradrenergic traits in the principal neurons and small intensely fluorescent cells of the parasympathetic sphenopalatine ganglion of the rat. Dev Biol.

[156] Reissmann E, Ernsberger U, Francis-West PH, Rueger D, Brickell PM, Rohrer H (1996). Involvement of bone morphogenetic proteins-4 and-7 in the specification of the adrenergic phenotype in developing sympathetic neurons. Development (Cambridge, England).

[157] Schneider C, Wicht H, Enderich J, Wegner M, Rohrer H (1999). Bone morphogenetic proteins are required in vivo for the generation of sympathetic neurons. Neuron.

[158] Shah NM, Groves AK, Anderson DJ (1996). Alternative neural crest cell fates are instructively promoted by TGFb superfamily members. Cell.

[159] Patzke H, Reissmann E, Stanke M, Bixby JL, Ernsberger U (2001). BMP growth factors and Phox2 transcription factors can induce synaptotagmin I and neurexin I during sympathetic neuron development. Mech Dev.

[160] Patzke H, Ernsberger U (2000). Expression of neurexin Ia splice variants in sympathetic neurons: selective changes during differentiation and in response to neurotrophins. Mol Cell Neurosci.

[161] Ross BS, Conroy WG (2008). Capabilities of neurexins in the chick ciliary ganglion. Dev Neurobiol.

[162] Lou X, Bixby JL (1993). Coordinate and noncoordinate regulation of synaptic vesicle protein genes during embryonic development. Dev Biol.

[163] Plunkett JA, Baccus SA, Bixby JL (1998). Differential regulation of synaptic vesicle protein genes by target and synaptic activity. J Neurosci.

[164] Landmesser L, Pilar G (1972). The onset and development of transmission in the chick ciliary ganglion. J Physiol.

[165] Landmesser L, Pilar G (1974). Synaptic transmission and cell death during normal ganglion development. J Physiol.

[166] Kaiser S, Blank M, Berg DK (2002). Maturation of postsynaptic nicotinic structures on autonomic neurons requires innervation but not cholinergic transmission. Eur J Neurosci.

[167] Moss BL, Schuetze SM, Role LW (1989). Functional properties and developmental regulation of nicotinic acetylcholine receptors on embryonic chicken sympathetic neurons. Neuron.

[168] Moss BL, Role LW (1993). Enhanced ACh sensitivity is accompanied by changes in ACh receptor channel properties and segregation of ACh receptor subtypes on sympathetic neurons during innervation *in vivo*. J Neurosci.

[169] Devay P, McGehee DS, Yu CR, Role LW (1999). Target-specific control of nicotinic receptor expression at developing interneuronal synapses in chick. Nat Neurosci.

[170] Dyachuk V, Furlan A, Shahidi MK, Giovenco M, Kauka N, Konstantinidou C, Pachnis V, Memic F, Marklund U, Müller T (2014). Parasympathetic neurons originate from nerve-associated peripheral glial progenitors. Science.

[171] Espinosa-Medina I, Outin E, Picard CA, Chettouh Z, Dymecki S, Consalez GG, Coppola E, Brunet JF (2014). Neurodevelopment. Parasympathetic ganglia derive from Schwann cell precursors. Science.

[172] Pattyn A, Guillemot F, Brunet JF (2006). Delays in neuronal differentiation in Mash1/Ascl1 mutants. Dev Biol.

[173] Tsarovina K, Pattyn A, Stubbusch J, Müller F, Van der Wees J, Schneider C, Brunet JF, Rohrer H (2004). Essential role of Gata transcription factors in sympathetic neuron development. Development (Cambridge, England).

[174] Tsarovina K, Reiff T, Stubbusch J, Kurek D, Grosveld FG, Parlato R, Schutz G, Rohrer H (2010). The Gata3 transcription factor is required for the survival of embryonic and adult sympathetic neurons. J Neurosci.

[175] Dubreuil W, Hirsch MR, Jouve C, Brunet JF, Goridis C (2002). The role of Phox2b in synchronizing pan-neuronal and type-specific aspects of neurogenesis. Development (Cambridge, England).

[176] Hirsch MR, Glover JC, Dufour HD, Brunet JF, Goridis C (2007). Forced expression of Phox2 homeodomain transcription factors induces a branchio-visceromotor axonal phenotype. Dev Biol.

[177] Song MR, Shirasaki R, Cai CL, Ruiz EC, Evans SM, Lee SK, Pfaff SL (2006). T-Box transcription factor Tbx20 regulates a genetic program for cranial motor neuron cell body migration. Development (Cambridge, England).

[178] Lichtman JW (1977). The reorganization of synaptic connexions in the rat submandibular ganglion during post-natal development. J Physiol (London).

[179] Sheu SH, Tapia JC, Tsuriel S, Lichtman JW (2017). Similar synapse elimination motifs at successive relays in the same efferent pathway during development in mice. Elife.

[180] Johnson DA, Purves DA (1981). Post-natal reduction of neural unit size in the rabbit ciliary ganglion. J Physiol (London).

[181] Purves, D, Lichtman, JW. The elimination of redundant preganglionic innervation to hamster sympathetic ganglion cells in early post-natal life. J Physiol (London). 1980;301:213–28. 10.1113/jphysiol.1980.sp013200 PMC1279393 7411428

[182] Snider WD (1987). The dendritic complexity and innervation of submandibular neurons in five species of mammals. J Neurosci.

[183] Forehand CJ, Purves D (1984). Regional innervation of rabbit ciliary ganglion cells by the terminals of preganglionic axons. J Neurosci.

[184] Purves D, Wigston DJ (1983). Neural units in the superior cervical ganglion of the Guinea pig. J Physiol (London).

[185] Nja A, Purves D (1977). Specific innervation of guinea-pig superior cervical ganglion cells by preganglionic fibres arising from different levels of the spinal cord. J Physiol.

[186] Nja A, Purves D (1978). The effects of nerve growth factor and its antiserum on synapses in the superior cervical ganglion of the Guinea-pig. J Physiol (London).

[187] Sharma N, Deppmann CD, Harrington AW, St Hillaire C, Chen ZY, Lee FS, Ginty DD (2010). Long-distance control of synapse assembly by target-derived NGF. Neuron.

[188] Causing CG, Gloster A, Aloyz R, Bamji SX, Chang E, Fawcett J, Kuchel G, Miller FD (1997). Synaptic innervation density is regulated by neuron-derived BDNF. Neuron.

[189] Singh KK, Miller FD (2005). Activity regulates positive and negative neurotrophin-derived signals to determine axon competition. Neuron.

[190] Singh KK, Park KJ, Hong EJ, Kramer BM, Greenberg ME, Kaplan DR, Miller FD (2008). Developmental axon pruning mediated by BDNF-p75NTR-dependent axon degeneration. Nat Neurosci.

[191] Majdazari A, Stubbusch J, Muller CM, Hennchen M, Weber M, Deng CX, Mishina Y, Schutz G, Deller T, Rohrer H (2013). Dendrite complexity of sympathetic neurons is controlled during postnatal development by BMP signaling. J Neurosci.

[192] Arber S (2012). Motor circuits in action: specification, connectivity, and function. Neuron.

[193] Chen H, He Z, Bagri A, Tessier-Lavigne M (1998). Semaphorin-neuropilin interactions underlying sympathetic axon responses to class III semaphorins. Neuron.

[194] Kawasaki T, Bekku Y, Suto F, Kitsukawa T, Taniguchi M, Nagatsu I, Nagatsu T, Itoh K, Yagi T, Fujisawa H (2002). Requirement of neuropilin 1-mediated Sema3A signals in patterning of the sympathetic nervous system. Development (Cambridge, England).

[195] Long JB, Jay SM, Segal SS, Madri JA (2009). VEGF-A and Semaphorin3A: modulators of vascular sympathetic innervation. Dev Biol.

[196] Marko SB, Damon DH (2008). VEGF promotes vascular sympathetic innervation. Am J Physiol Heart Circ Physiol.

[197] Makita T, Sucov HM, Gariepy CE, Yanagisawa M, Ginty DD (2008). Endothelins are vascular-derived axonal guidance cues for developing sympathetic neurons. Nature.

[198] Manousiouthakis E, Mendez M, Garner MC, Exertier P, Makita T (2014). Venous endothelin guides sympathetic innervation of the developing mouse heart. Nat Commun.

[199] Brunet I, Gordon E, Han J, Cristofaro B, Broqueres-You D, Liu C, Bouvree K, Zhang J, del Toro R, Mathivet T (2014). Netrin-1 controls sympathetic arterial innervation. J Clin Invest.

[200] Nilsson S. Autonomic Nerve Function in the Vertebrates; Zoophysiology Vol. 13: Spinger-Verlag, Berlin Heidelberg New York; 1983.

[201] Gibbins I (2013). Functional organization of autonomic neural pathways. Organogenesis.

[202] Jänig W, Keast JR, McLachlan EM, Neuhuber WL, Southard-Smith EM (2016). Science.

[203] Janig W, Keast JR, McLachlan EM, Neuhuber WL, Southard-Smith M (2017). Renaming all spinal autonomic outflows as sympathetic is a mistake. Auton Neurosci.

[204] Janig W, McLachlan EM, Neuhuber WL (2018). The sacral autonomic outflow: against premature oversimplification. Clin Auton Res.

[205] Horn JP, de Groat WC (2016). Functional criteria define divisions of the autonomic motor system. Science.

[206] Espinosa-Medina I, Saha O, Boismoreau F, Brunet JF (2018). The “sacral parasympathetic”: ontogeny and anatomy of a myth. Clin Auton Res.

[207] Fritzsch B, Elliott KL, Glover JC (2017). Gaskell revisited: new insights into spinal autonomics necessitate a revised motor neuron nomenclature. Cell Tissue Res.

[208] Horn JP (2018). The sacral autonomic outflow is parasympathetic: Langley got it right. Clin Auton Res.

[209] Neuhuber W, McLachlan E, Janig W (2017). The sacral autonomic outflow is spinal, but not “sympathetic”. Anat Rec.

[210] Deneris ES, Hobert O (2014). Maintenance of postmitotic neuronal cell identity. Nat Neurosci.

[211] Flames N, Hobert O (2011). Transcriptional control of the terminal fate of monoaminergic neurons. Annu Rev Neurosci.

[212] Thaler JP, Koo SJ, Kania A, Lettieri K, Andrews S, Cox C, Jessell TM, Pfaff SL (2004). A postmitotic role for Isl-class LIM homeodomain proteins in the assignment of visceral spinal motor neuron identity. Neuron.

[213] Spyer KM, Gourine AV (2009). Chemosensory pathways in the brainstem controlling cardiorespiratory activity. Philos Trans R Soc Lond Ser B Biol Sci.

[214] Taylor EW, Jordan D, Coote JH (1999). Central control of the cardiovascular and respiratory systems and their interactions in vertebrates. Physiol Rev.

[215] DiBona GF (2005). Physiology in perspective: the wisdom of the body. Neural control of the kidney. Am J Physiol Regul Integr Comp Physiol.

[216] DiBona GF (2005). Dynamic analysis of patterns of renal sympathetic nerve activity: implications for renal function. Exp Physiol.

[217] Boudoulas KD, Triposkiadis F, Parissis J, Butler J, Boudoulas H (2017). The cardio-renal interrelationship. Prog Cardiovasc Dis.

[218] Jänig W, Häbler H-J (2003). Neurophysiological analysis of target-related sympathetic pathways - from animal to human: similarities and differences. Acta Physiol Scand.

[219] Jänig W, McLachlan EM (1992). Characteristics of function-specific pathways in the sympathetic nervous system. Trends Neurosci.

[220] Lichtman JW, Purves D, Yip JW (1979). On the purpose of selective innervation of guinea pig superior cervical ganglion cells. J Physiol (London).

[221] Varley JE, Wehby RG, Rueger DC, Maxwell GD (1995). Number of adrenergic and Islet-1 Immunoreactive cells is increased in avian trunk neural crest cultures in the presence of human recombinant osteogenic Protein-1. Dev Dyn.

[222] Stanke M, Geissen M, Götz R, Ernsberger U, Rohrer H (2000). The early expression of VAChT and VIP in mouse sympathetic ganglia is not induced by cytokines acting through LIFRb or CNTFRa. Mech Dev.

[223] Morikawa Y, D'Autreaux F, Gershon MD, Cserjesi P (2007). Hand2 determines the noradrenergic phenotype in the mouse sympathetic nervous system. Dev Biol.

[224] Hendershot TJ, Liu H, Clouthier DE, Shepherd IT, Coppola E, Studer M, Firulli AB, Pittman DL, Howard MJ (2008). Conditional deletion of Hand2 reveals critical functions in neurogenesis and cell type-specific gene expression for development of neural crest-derived noradrenergic sympathetic ganglion neurons. Dev Biol.

[225] Moriguchi T, Takako N, Hamada M, Maeda A, Fujioka Y, Kuroha T, Huber RE, Hasegawa SL, Rao A, Yamamoto M (2006). Gata3 participates in a complex transcriptional feedback network to regulate sympathoadrenal differentiation. Development (Cambridge, England).

[226] Howard MJ, Stanke M, Schneider C, Wu X, Rohrer H (2000). The transcription factor dHAND is a downstream effector of BMPs in sympathetic neuron specification. Development (Cambridge, England).

[227] Osumi N, Hirota A, Ohuchi H, Nakafuku M, Iimura T, Kuratani S, Fujiwara M, Noji S, Eto K (1997). Pax-6 is involved in the specification of hindbrain motor neuron subtype. Development (Cambridge, England).

[228] Pattyn A, Morin X, Cremer H, Goridis C, Brunet JF (1997). Expression and interactions of the two closely related homeobox genes *Phox2a* and *Phox2b* during neurogenesis. Development (Cambridge, England).

[229] Dubreuil V, Hirsch MR, Pattyn A, Brunet JF, Goridis C (2000). The Phox2b transcription factor coordinately regulates neuronal cell cycle exit and identity. Development (Cambridge, England).

[230] Zhou Q, Anderson DJ (2002). The bHLH transcription factors OLIG2 and OLIG1 couple neuronal and glial subtype specification. Cell.

[231] Lu QR, Sun T, Zhu Z, Ma N, Garcia M, Stiles CD, Rowitch DH (2002). Common developmental requirement for Olig function indicates a motor neuron/oligodendrocyte connection. Cell.

[232] Erselius JR, Goulding MD, Gruss P (1990). Structure and expression pattern of the murine Hox-3.2 gene. Development (Cambridge, England).

[233] Liu JP, Laufer E, Jessell TM (2001). Assigning the positional identity of spinal motor neurons: Rostrocaudal patterning of Hox-c expression by FGFs, Gdf11, and retinoids. Neuron.

[234] Dasen JS, Liu J-P, Jessell TM (2003). Motor neuron columnar fate imposed by sequential phases of Hox-c activity. Nature.

[235] Jung H, Lacombe J, Mazzoni EO, Liem KF, Grinstein J, Mahony S, Mukhopadhyay D, Gifford DK, Young RA, Anderson KV (2010). Global control of motor neuron topography mediated by the repressive actions of a single hox gene. Neuron.

[236] Dasen JS, De Camilli A, Wang B, Tucker PW, Jessell TM (2008). Hox repertoires for motor neuron diversity and connectivity gated by a single accessory factor, FoxP1. Cell.

[237] Kraus F, Haenig B, Kispert A (2001). Cloning and expression analysis of the mouse T-box gene tbx20. Mech Dev.

[238] Rousso DL, Gaber ZB, Wellik D, Morrisey EE, Novitch BG (2008). Coordinated actions of the forkhead protein Foxp1 and Hox proteins in the columnar organization of spinal motor neurons. Neuron.

[239] Varela-Echavarria A, Pfaff SL, Guthrie S (1996). Differential expression of LIM homeobox genes among motor neuron subpopulations in the developing chick brain stem. Mol Cell Neurosci.

[240] Ericson J, Rashbass P, Schedl A, Brenner-Morton S, Kawakami A, Van Heyningen V, Jessell TM, Briscoe J (1997). Pax6 controls progenitor cell identity and neuronal fate in response to graded shh signaling. Cell.

[241] George KM, Leonard MW, Roth ME, Lieuw KH, Kioussis D, Grosveld F, Engel JD (1994). Embryonic expression and cloning of the murine GATA-3 gene. Development (Cambridge, England).

[242] Lim K-C, Lakshmanan G, Crawford SE, Gu Y, Grosveld F, Engel JD (2000). Gata3 loss leads to embryonic lethality due to noradrenaline deficiency of the sympathetic nervous system. Nat Genet.

[243] Doxakis E, Howard L, Rohrer H, Davies AM (2008). HAND transcription factors are required for neonatal sympathetic neuron survival. EMBO Rep.

[244] Howard M, Foster DN, Cserjesi P (1999). Expression of *Hand* gene products may be sufficient for the differentiation of avian neural crest-derived cells into catecholaminergic neurons in culture. Dev Biol.

[245] Vincentz JW, VanDusen NJ, Fleming AB, Rubart M, Firulli BA, Howard MJ, Firulli AB (2012). A Phox2- and Hand2-dependent Hand1 cis-regulatory element reveals a unique gene dosage requirement for Hand2 during sympathetic neurogenesis. J Neurosci.

[246] Wang W, Lo P, Frasch M, Lufkin T (2000). Hmx: an evolutionary conserved homeobox gene family expressed in the developing nervous system in mice and drosophila. Mech Dev.

